# Supplementary material for: Multiple independent losses of the biosynthetic pathway for two tropane alkaloids in the Solanaceae family
Source: Nat Commun. 2023 Dec 20;14:8457. doi: 10.1038/s41467-023-44246-3 (PMC10730914; doi:10.1038/s41467-023-44246-3)
Supplement: Supplementary file 1 — Supplementary Information [file 41467_2023_44246_MOESM1_ESM.pdf]

Multiple independent losses of the biosynthetic pathway for two tropane alkaloids in the Solanaceae family

Yang *et al.*

Supplementary Method 1. Phylogenetic analyses of the Solanaceae species based on three data sets

The species tree of the 11 Solanaceae species was constructed based on three datasets: chloroplast genomes, single-copy nuclear genes and BUSCO conserved genes. The organelle chloroplast genomes of *B. arborea* and *M. caulescens* were assembled using GetOrganelle[1], while the chloroplast genomes of *P. axillaris*, *N. attenuata*, *L. chinense*, *Atropa belladonna*, *Hyoscyamus niger*, *A. tangutica*, *S. melongena*, *Solanum lycopersicum*, *S. tuberosum*, and *Arabidopsis thaliana* were obtained from the NCBI database (http://www.ncbi.nlm.nih.gov) with the corresponding accession numbers (MF459662.1, NC_035952.1, NC_042204.1, NC_004561.1, KF248009.1, MK347419.1, MF818319.1, NC_007898.3, DQ231562.1, KX551970.1). For phylogenetic analysis of Solanaceae and Convolvulaceae, the *ndhF*, *matK*, trans/G, trnL/F sequences were download from NCBI database. Multiple sequence alignments for all sequences were performed using MAFFT[2] with default parameters. The chloroplast phylogenetic tree was constructed using RAxML[3] based on the concatenated sequences of the 11 species. For the single-copy nuclear genes dataset, orthologous gene groups were identified in 12 species using the OrthoMCL program[4]. Multiple sequence alignments for these sequences were performed using MAFFT[6] with default parameters. A total of 270 single-copy genes and 257 BUSCO conserved genes were used to construct species tree.

Supplementary Method 2. Weighted gene co-expression network analysis (WGCNA)

To comprehensively investigate the co-expression patterns of genes related to the HS pathway, we employed weighted gene co-expression networks (WGCNA) using all differential expressed genes in *A. tanguticus*. Plants samples of *A. tanguticus* were collected from Menyuan (Qinghai Province), China, and subsequently transplanted to Botanical Garden, College of Ecology, Lanzhou University, Lanzhou, China. Various plant tissues, including mature leaves, juvenile leaves, stems, root and secondary root, were harvested and immediately frozen and stored at -80 °C for RNA sequencing (RNAseq). For transcriptome atlases of these five tissues, WGCNA analysis was performed on the dataset of differentially expressed genes (DEGs) using the R package WGCNA version 1.68[5]. Each tissue consisted of three biological replicates and gene expression was transformed to log2 (TPM + 1) values. Each dataset underwent network analysis with a soft threshold (power/β) determined to produce a scale-free network with optimal scale-free topology model fit and mean connectivity. Next, we utilized the WGCNA blockwise Modules function to construct a signed network. In short, gene co-expression relationships were calculated as bi-weight mid-correlation coefficients raised to the soft threshold, transforming the gene expression correlation adjacency matrix into a TOM (topological overlap matrix), which was then converted to a dissimilarity matrix that was used to generate a hierarchical cluster tree. To identify the co-expressed gene modules, we employed the dynamic tree cut method with the following parameters: deepSplit level 2, detectCutHeight of 0.995, minModuleSize of 30, and tree mergeCutHeight of 0.25.

To investigate the gene expression patterns of the consensus modules across samples, we represented each module by a module eigengene (*ME*). *ME* was calculated as the first principle component of the expression profiles of each module. Furthermore, we calculated the connectivity of each gene to its corresponding module using a module membership (*kME*) value that was defined as the bi-weight mid-correlation between the gene expression and its corresponding *ME*[5]. By utilizing the intramodular *kME* values of each gene, we identified hub genes within the network[5,6]. To explore the relationships between co-expression modules, we constructed an eigengene network. This network was established based on the bi-weight mid-correlation between the module eigengenes, providing insights into the interplay between different co-expression modules[7].

Overall, we successfully identified 10 modules in *A. tanguticus* (Supplementary Figure 37). Notably, the yellow modules exhibited a high secondary root tissues expression pattern. These yellow modules encompassed genes associated with the hyoscyamine and scopolamine (HS) biosynthetic pathway, such as *PMT*, *MPO*, *PYKS*, *HDH*, and *H6H* in *A. tanguticus*. The visualization of network was executed using Cytoscape[8] (v 3.6.0). These findings collectively demonstrate that the genes involved in the HS biosynthesis pathway have been extensively characterized in *A. tanguticus*.

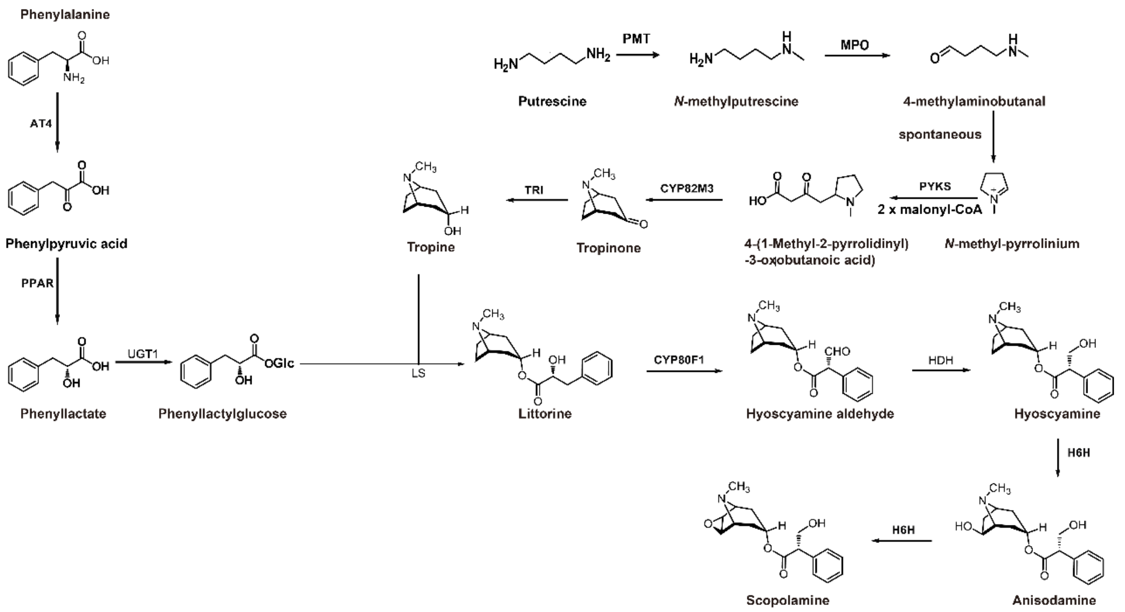

Supplementary Figure 1. Overview of HS biosynthetic pathway.

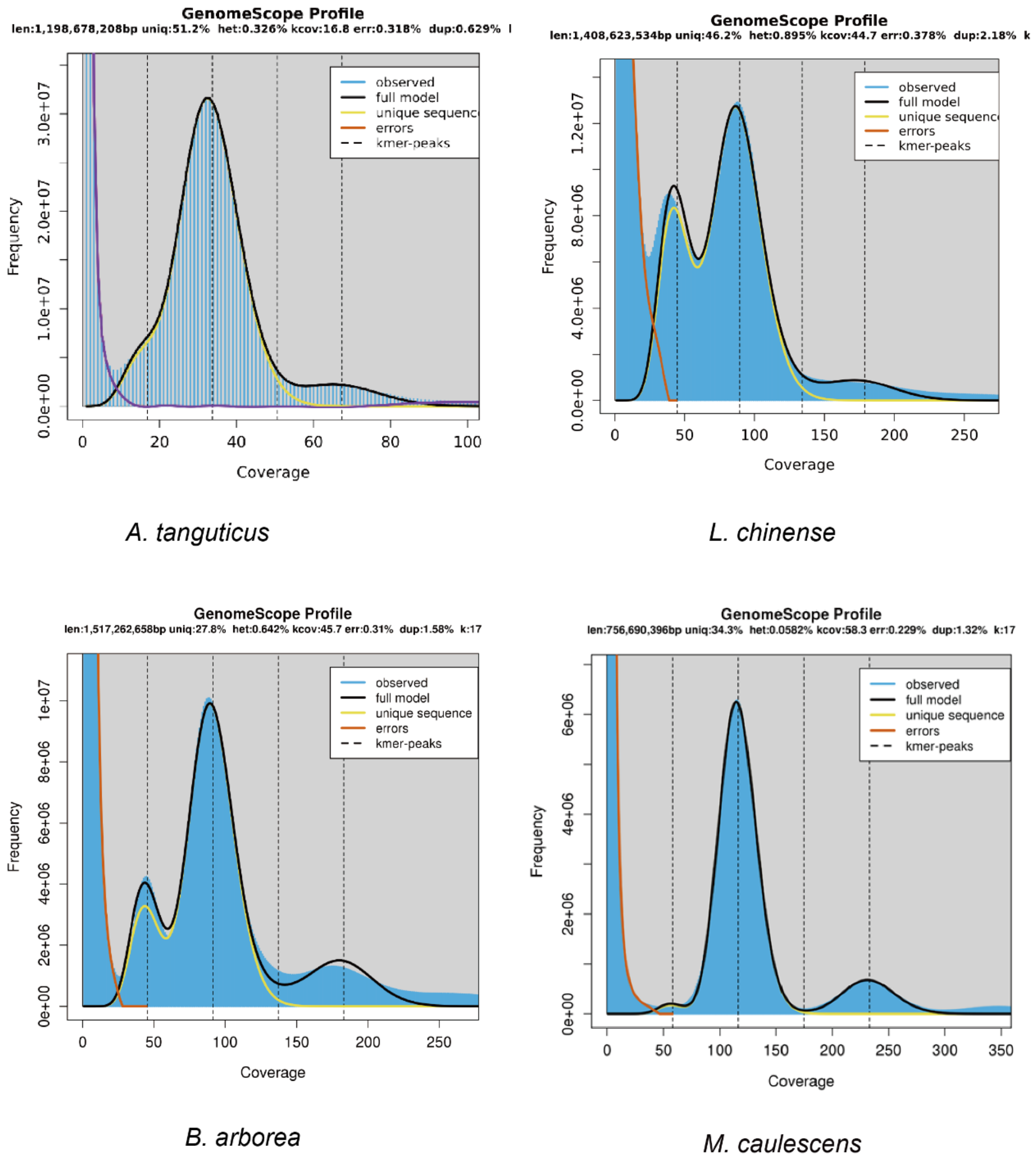

Supplementary Figure 2. Genome size estimation by GenomeScope of *A. tanguticus*, *L. chinense*, *M. caulescens* and *B. arborea*. *K*-mer size was set as 17 and 23 and the default parameters were used within GenomeScope.

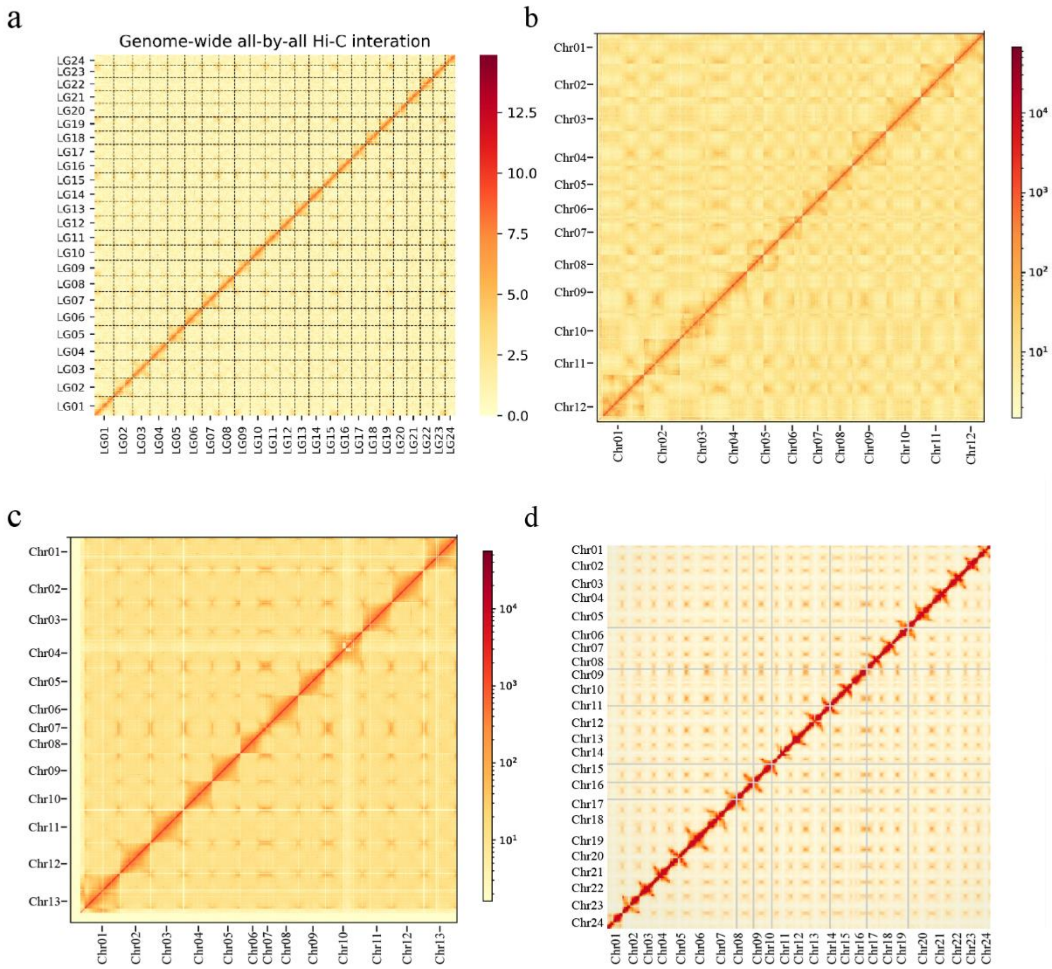

Supplementary Figure 3. Chromosome-level assembly interaction heat map. a, *A. tanguticus*. b, *L. chinense.* c, *B. arborea*. d, *M. caulescens.*

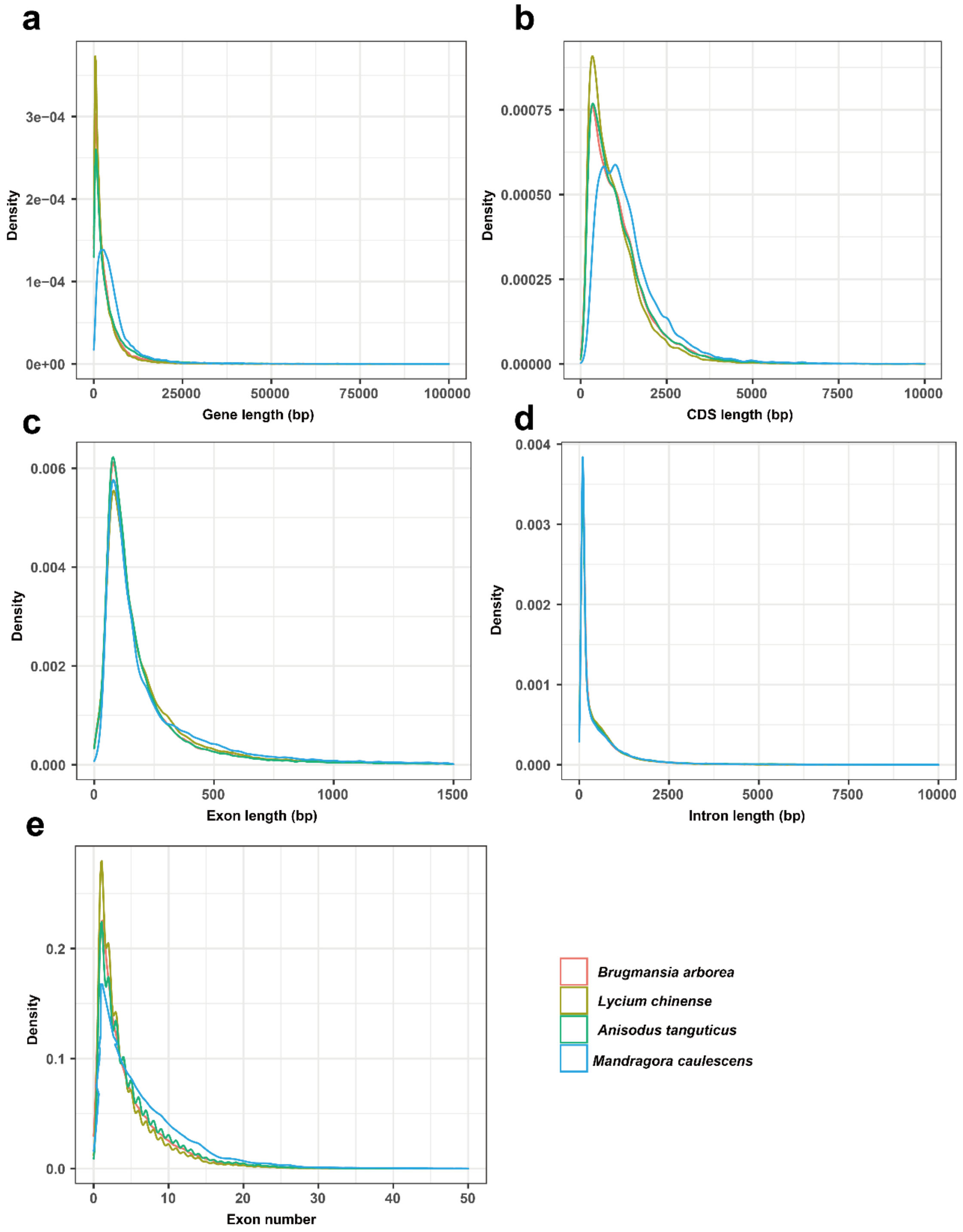

Supplementary Figure 4. Comparison of gene structure characteristics in *A. tanguticus*, *L. chinense, M. caulescens* and *B. arborea*. a, gene length. b, CDS length. c, exon length. d, intron length. e, exon number.

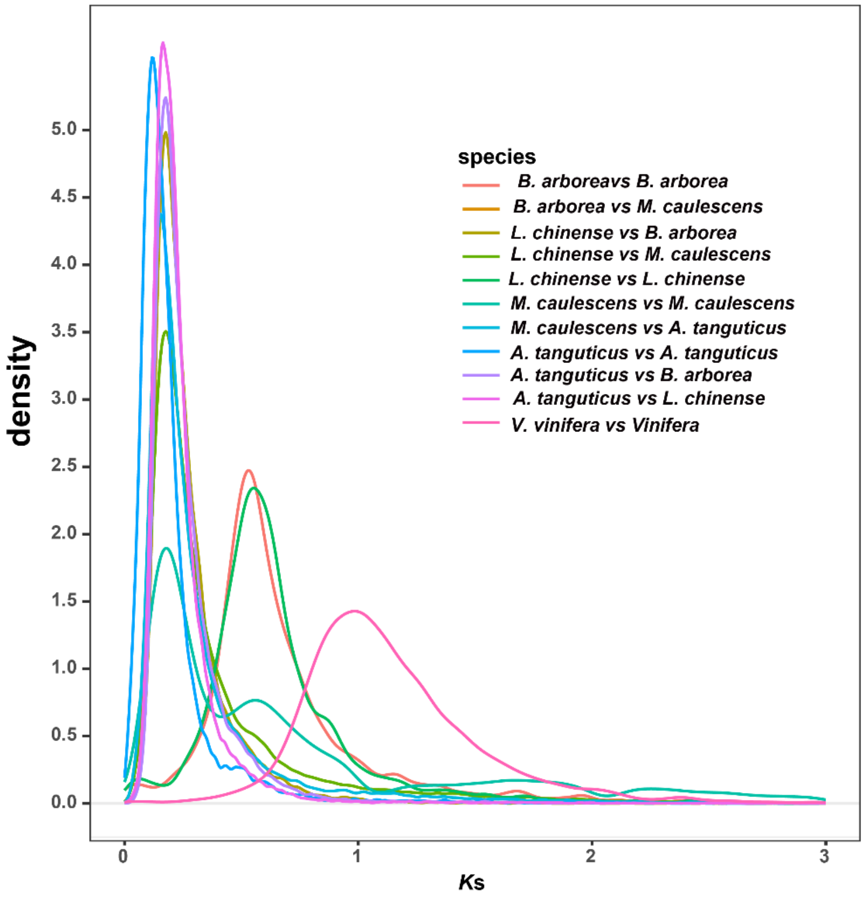

Supplementary Figure 5. The distribution of synonymous nucleotide substitutions (*K*s) of syntenic blocks. Syntenic blocks (involving ≥ five colinear genes) within one species or between two species were collected, and the median *K*s values of each block were obtained.

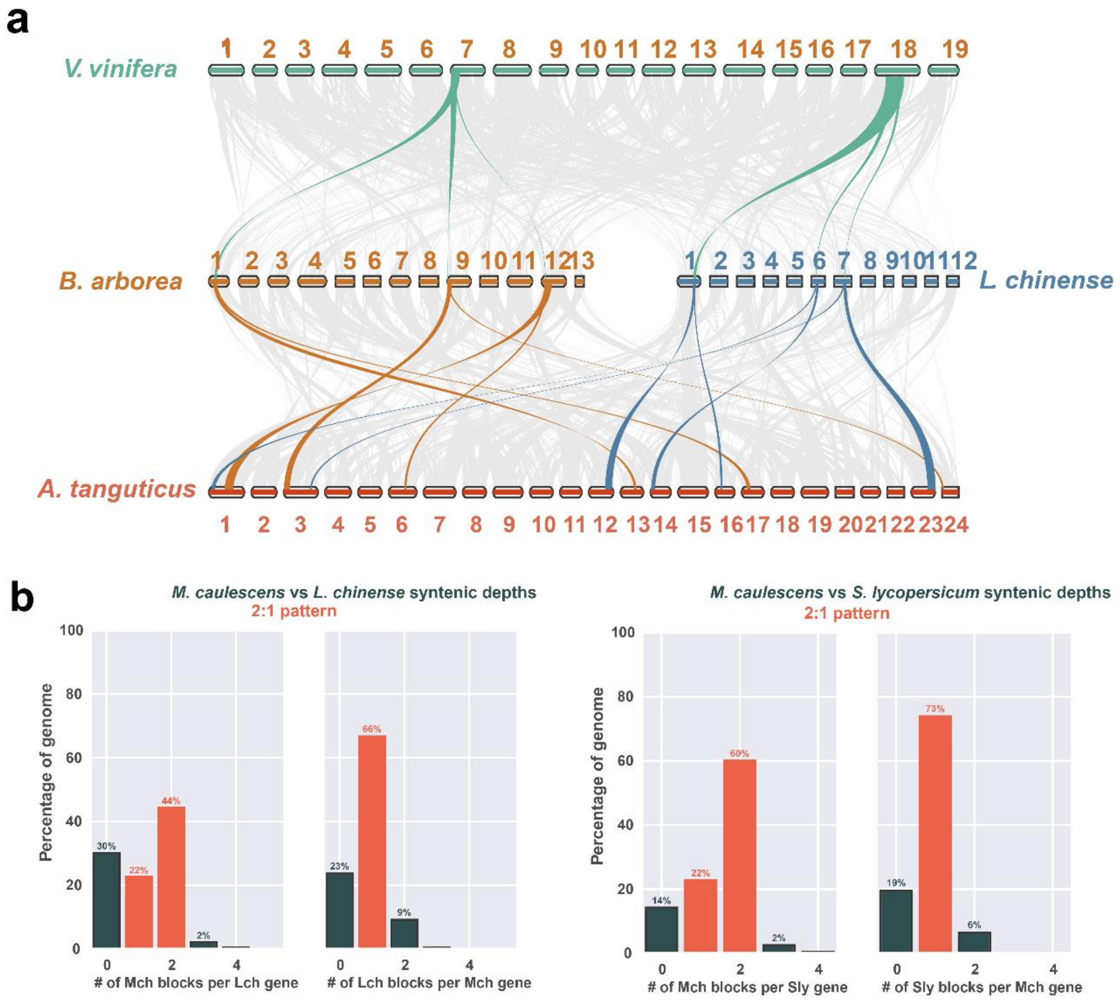

Supplementary Figure 6. Inter-genomic syntenic analysis of the *A. tanguticus*, *L. chinense*, *B. arborea*, *M. caulescens* and others genomes. a, A genomic region in *V. vinifera* could be aligned with up to three regions in *B. arborea* and *L. chinense* respectively. A genomic region in *B. arborea* and *L. chinense* could be aligned with up to two regions in *A. tanguticus* respectively. Examples of chromosomes are highlighted with colors. b, The genomic region in Lch (*L. chinense*) and *Sly* (*S. lycopersicum*) could be aligned with up to two regions in Mch (*M. caulescens*) respectively.

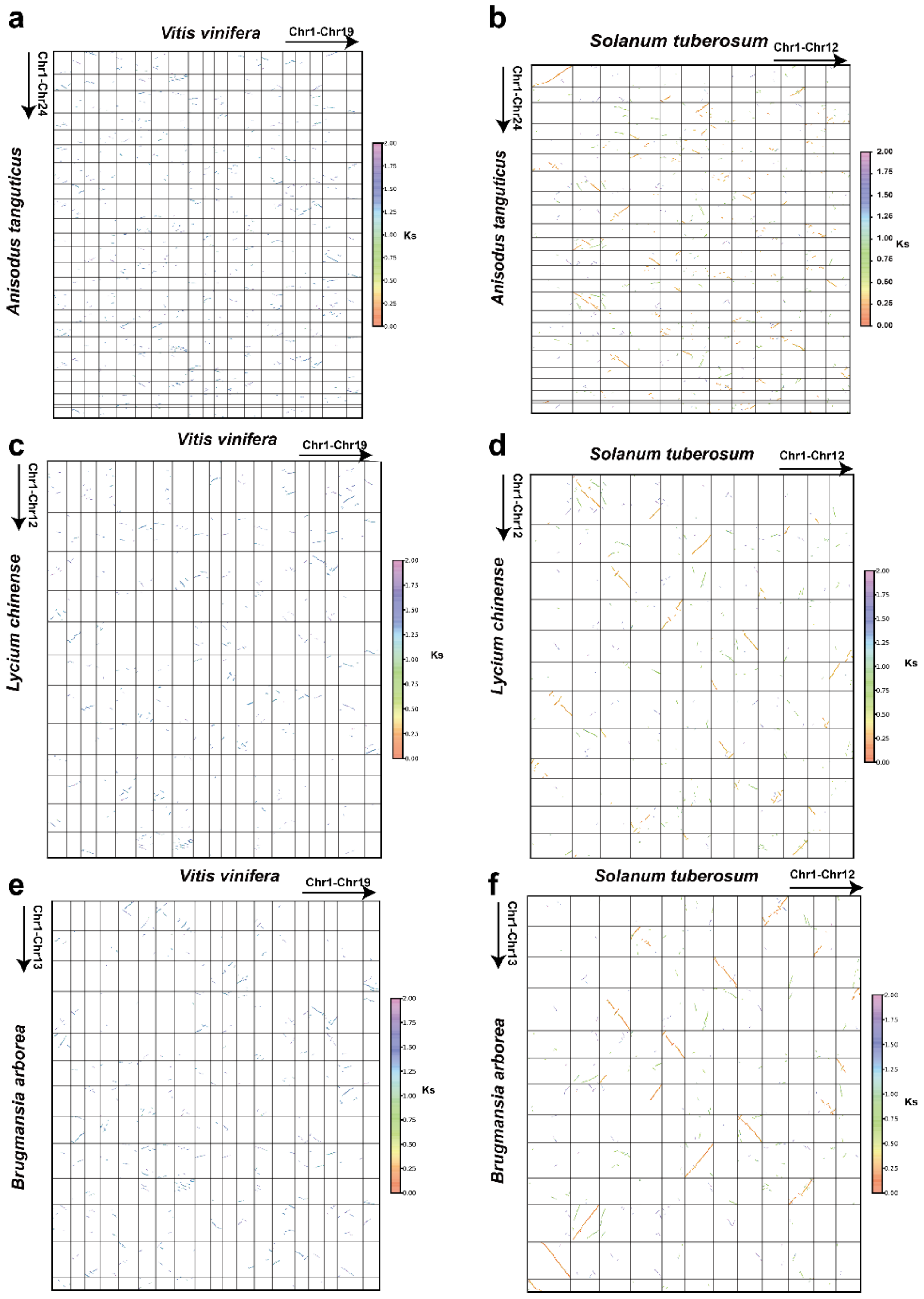

Supplementary Figure 7. Inter-genomic syntenic block dotplot between *A. tanguticus L. chinense* and *B. arborea* with *V. vinifera* and *S. tuberosum*, respectively. a, The syntenic block dotplot with *A. tanguticus* and *V. vinifera.* b, The syntenic block dotplot with *A. tanguticus* and *S. tuberosum.* c, The syntenic block dotplot with *L. chinense* and *V. vinifera.* d, The syntenic block dotplot with *L. chinense* and *S. tuberosum.* e, The syntenic block dotplot with *B. arborea* and *V. vinifera.* f, The syntenic block dotplot with *B. arborea* and *S. tuberosum.*

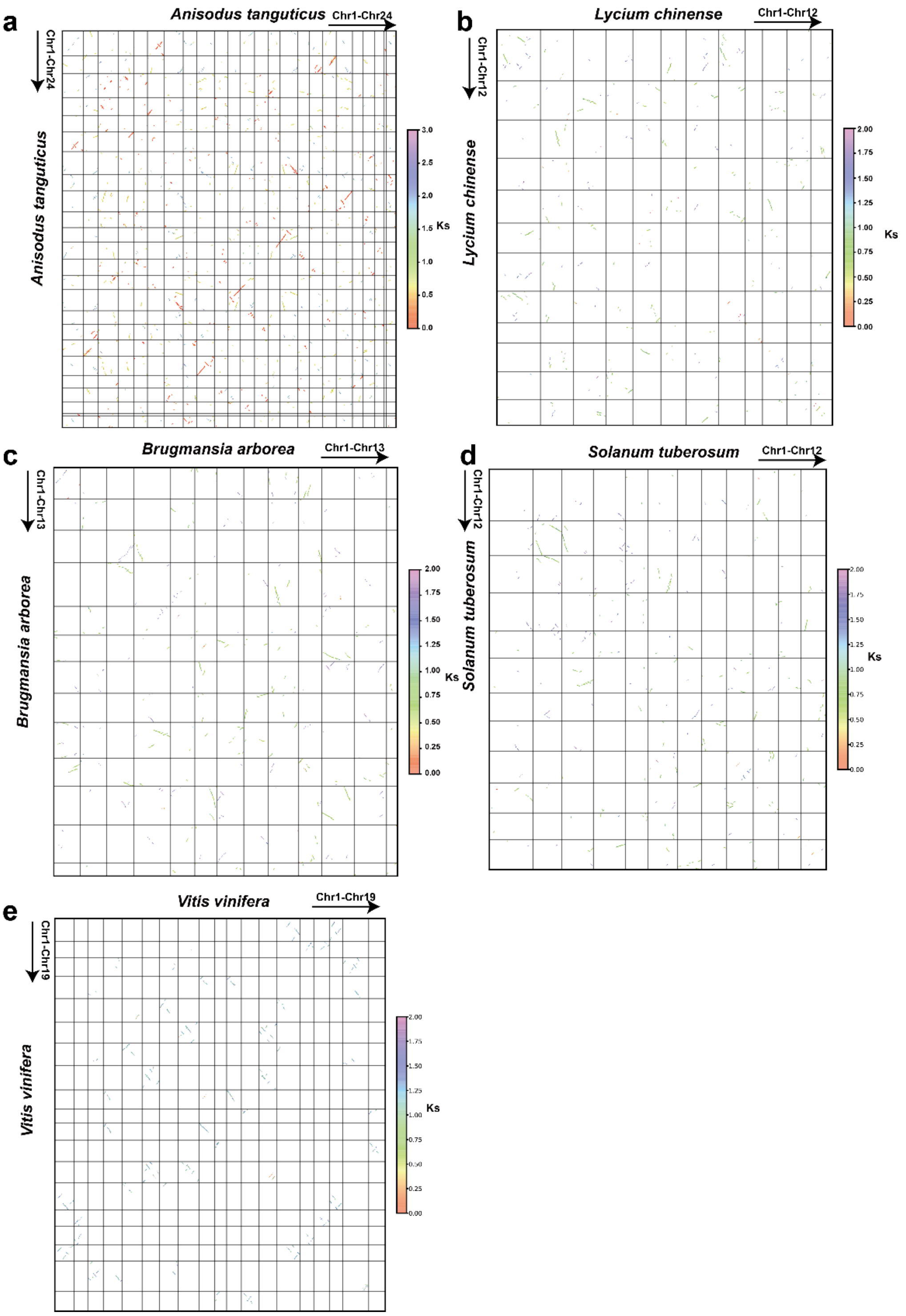

Supplementary Figure 8. Intra-genomic syntenic block dotplot of *A. tanguticus L. chinense*, *B. arborea*, *S. tuberosum* and *V. vinifera*. a, The syntenic block dotplot with *A. tanguticus* and *A. tanguticus.* b, The syntenic block dotplot with *L. chinense* and *L. chinense.* c, The syntenic block dotplot with *B. arborea* and *B. arborea.* d, The syntenic block dotplot with *S. tuberosum* and *S. tuberosum.* e, The syntenic block dotplot with *V. vinifera* and *V. vinifera.*

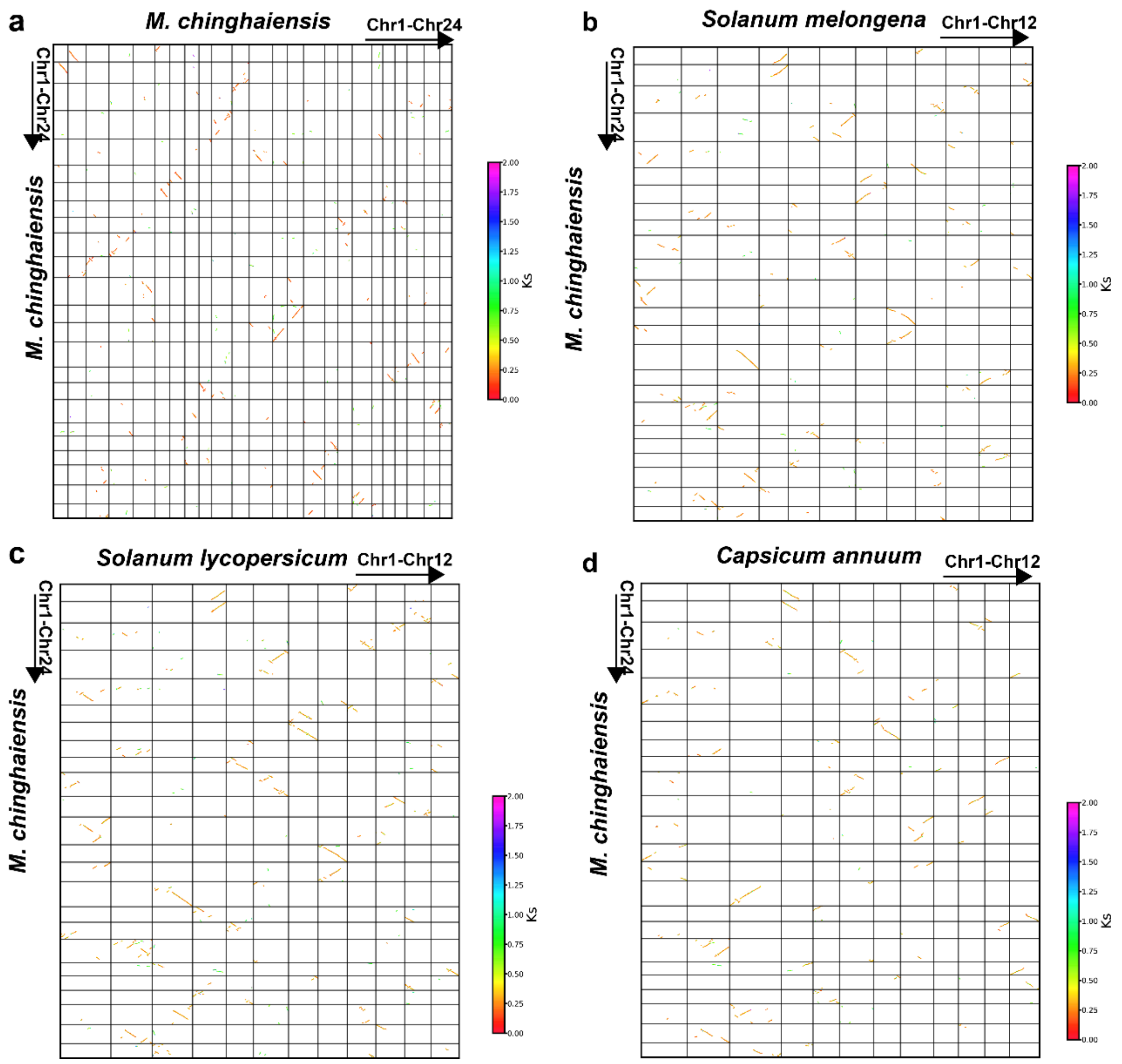

Supplementary Figure 9. Intra-genomic of *M. caulescens* and inter-genomic syntenic block dotplot between *M. caulescens* with *S. melongena, S. lycopericum* and *C. annuum*, respectively. a, The syntenic block dotplot with *M. caulescens* and *M. caulescens*. b, The syntenic block dotplot with *M. caulescens* ***a****nd S. melongena.* c, The syntenic block dotplot with *M. caulescens and S. lycopericum.* d, The syntenic block dotplot with *M. caulescens and C. annuum.*

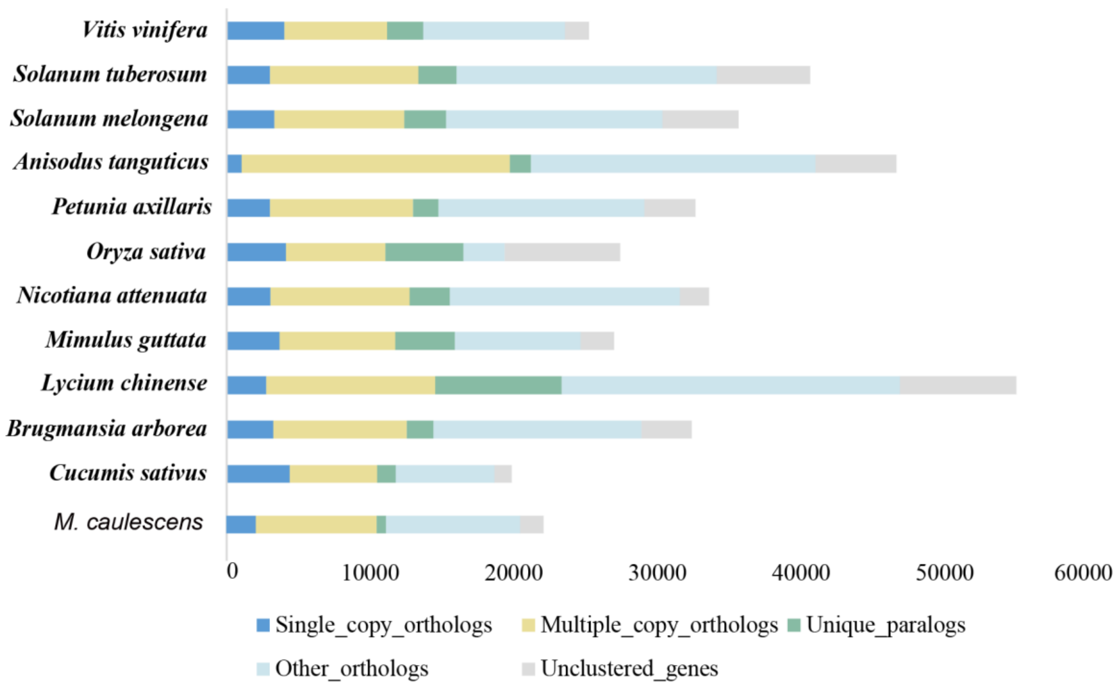

Supplementary Figure 10. The distribution of single-copy, multiple-copy, unique, other and total orthologs using OrthoMCL software in 12 species. Source data are provided as a Source Data file.

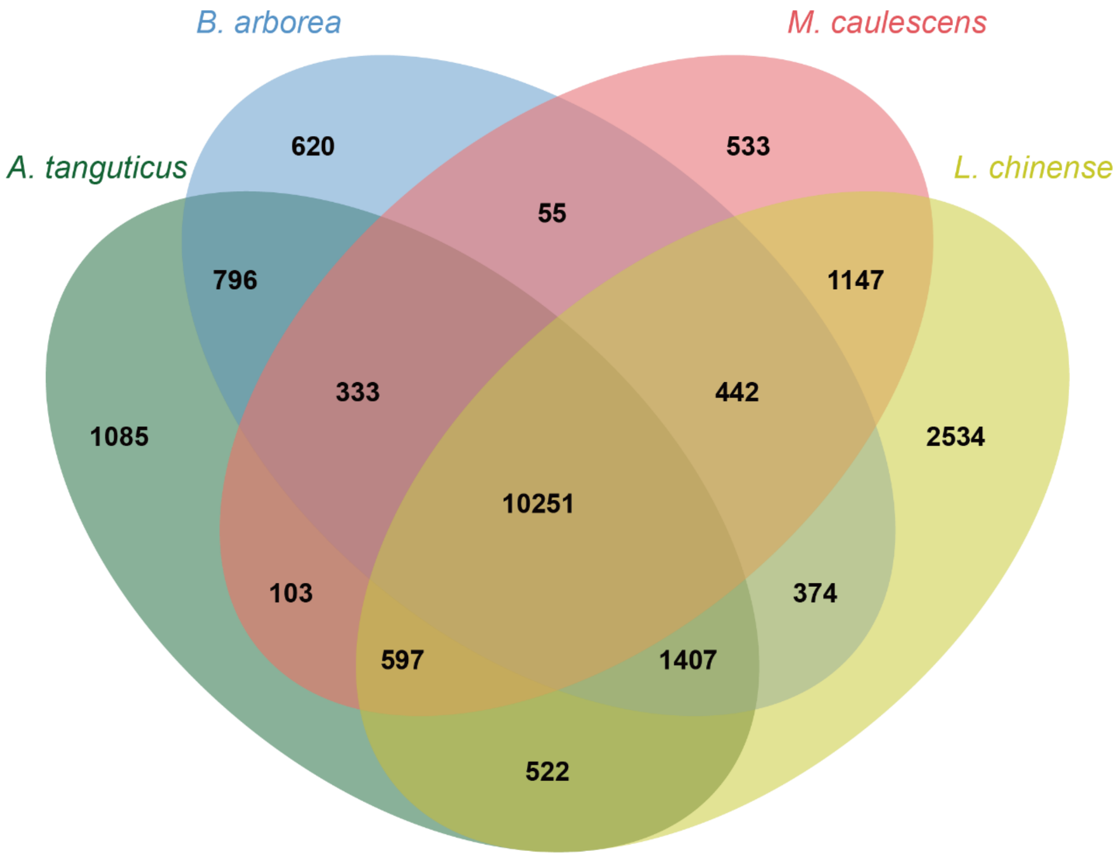

Supplementary Figure 11. Venn diagram of shared orthologous gene families in *A. tanguticus*, *L. chinense*, *B. arborea* and *M. caulescens*. The number of gene families is listed for each component. Source data are provided as a Source Data file.

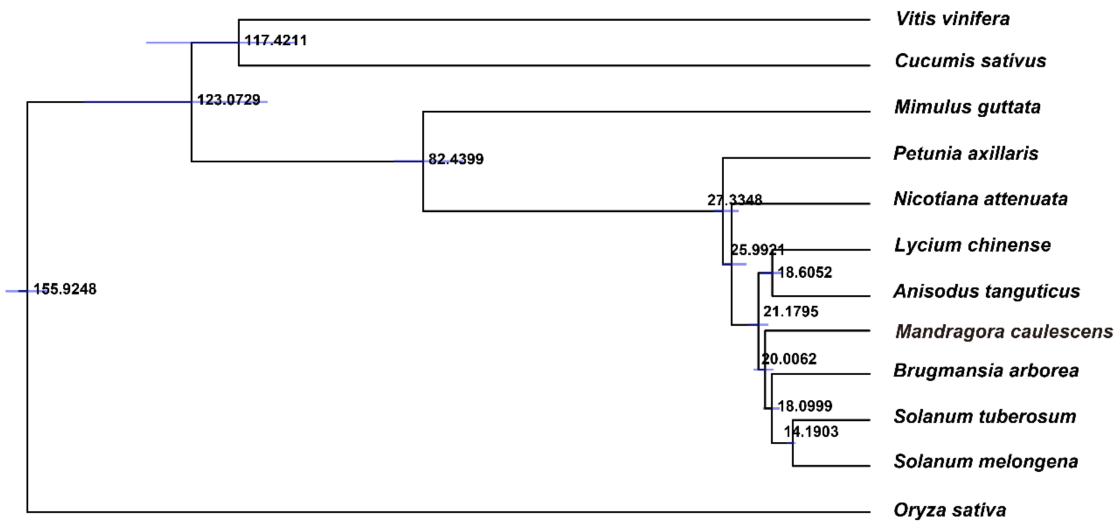

Supplementary Figure 12. Divergence time estimated among 12 species. Divergence estimates (Mya, million years ago) are indicated above each node and the blue nodal bars show the 95% confidence intervals. Source data are provided as a Source Data file.

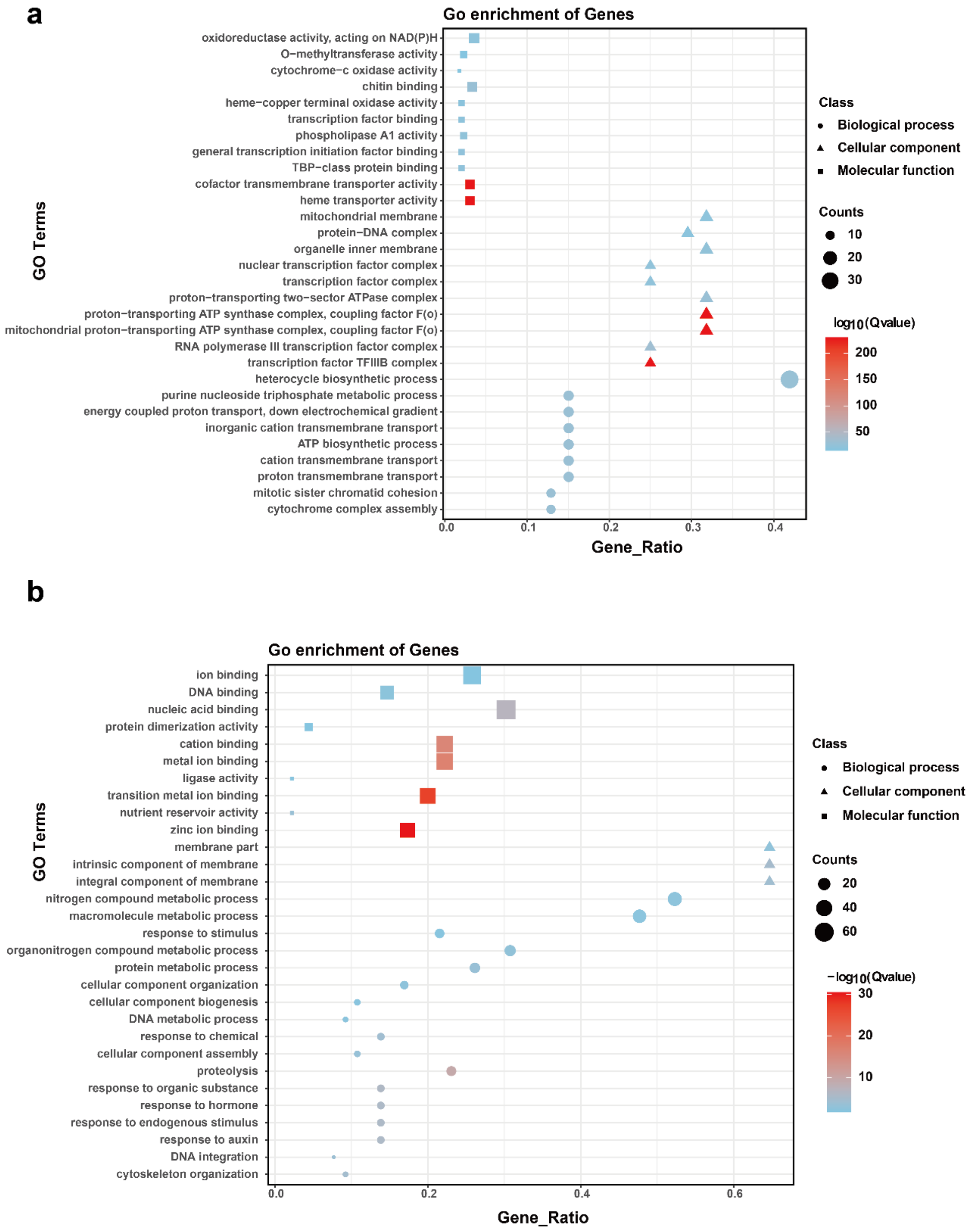

Supplementary Figure 13. The Gene Ontology (GO) enrichment analysis in *A. tanguticus*. a, The significantly enriched GO terms for genes with expansion. b, The significantly enriched GO terms for genes with unique. Source data are provided as a Source Data file.

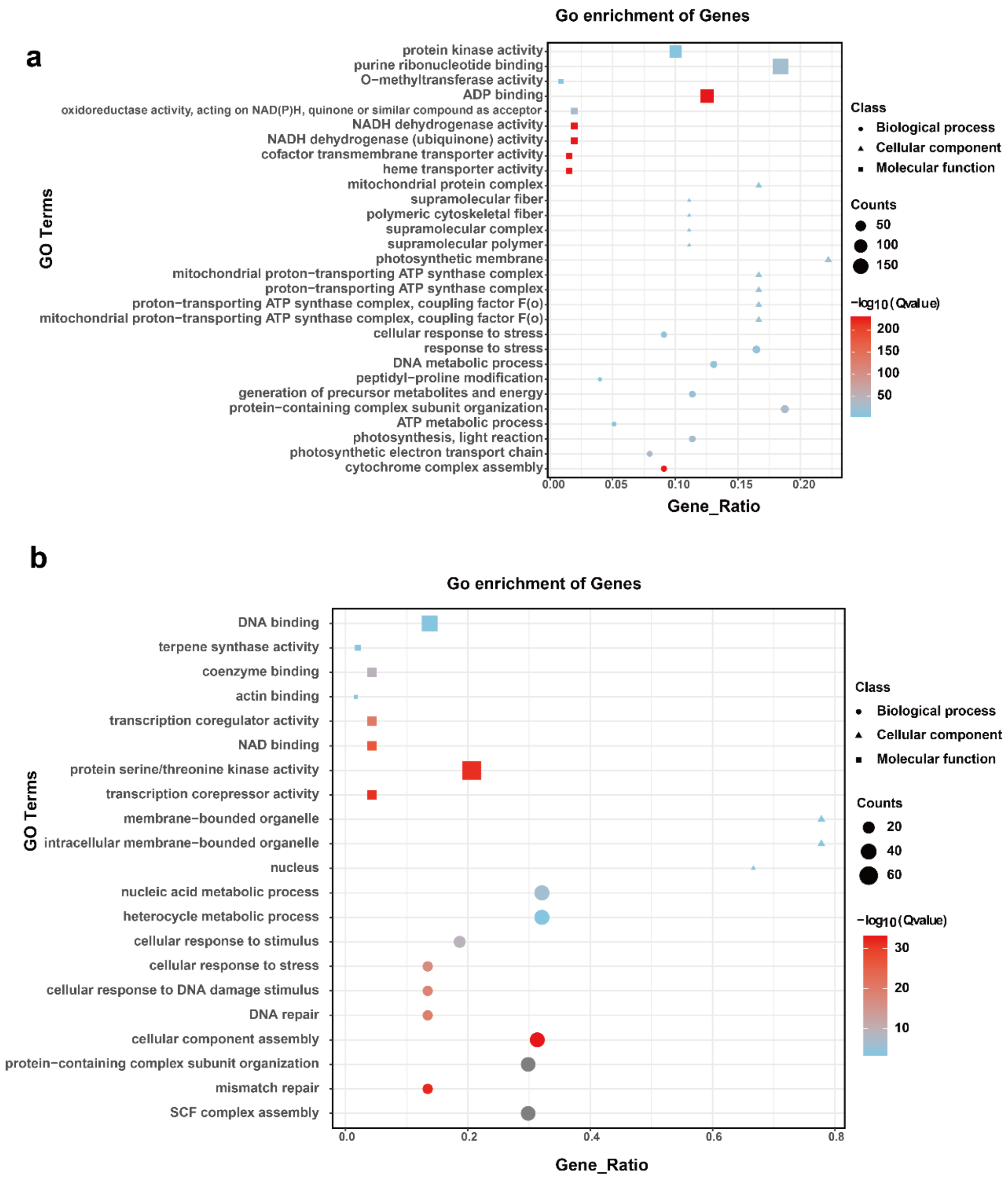

Supplementary Figure 14. The Gene Ontology (GO) enrichment analysis in *B. arborea*. a, The significantly enriched GO terms for genes with expansion. b, The significantly enriched GO terms for genes with unique. Source data are provided as a Source Data file.

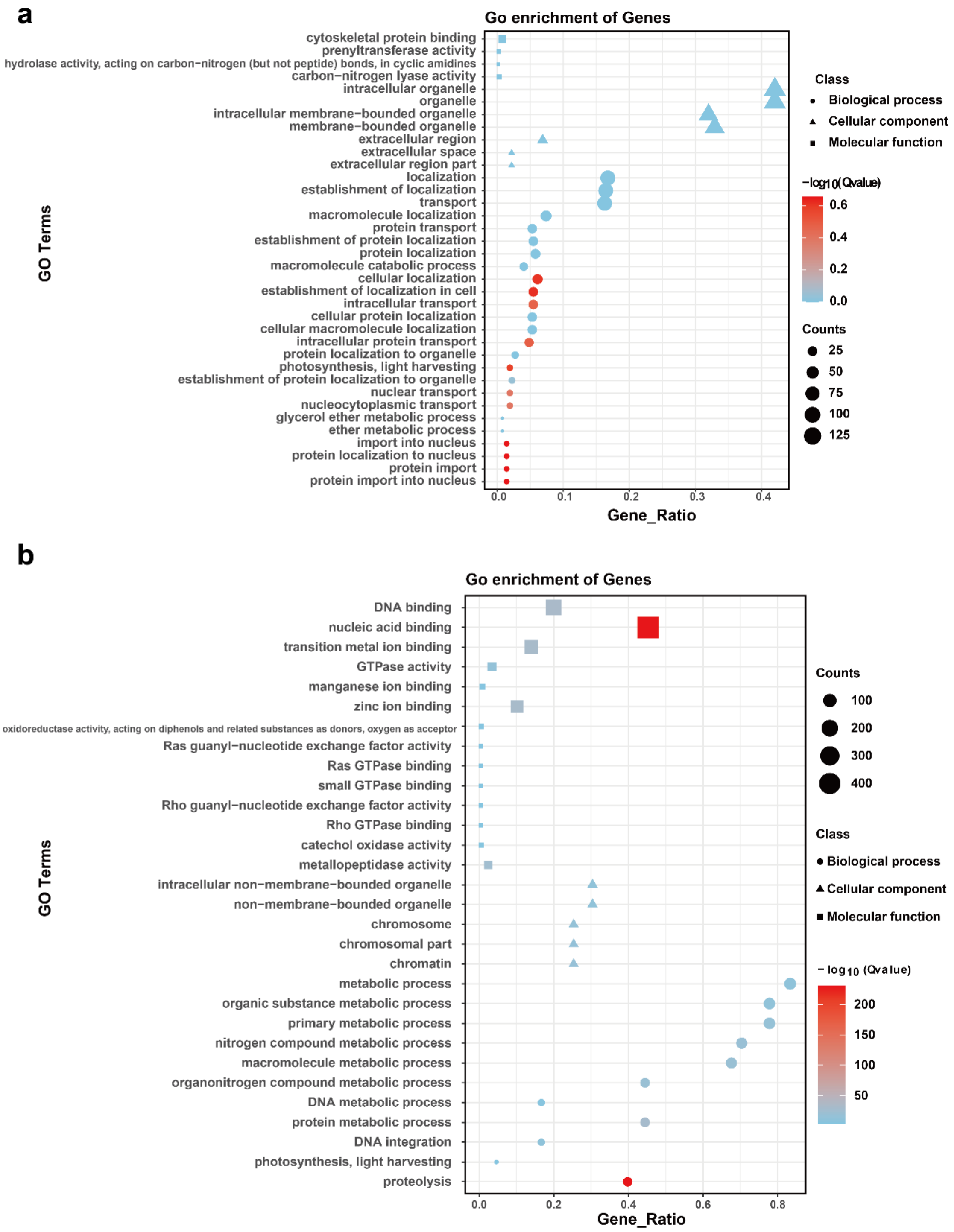

Supplementary Figure 15. The Gene Ontology (GO) enrichment analysis in *L. chinense*. a, The significantly enriched GO terms for genes with expansion. b, The significantly enriched GO terms for genes with unique. Source data are provided as a Source Data file.

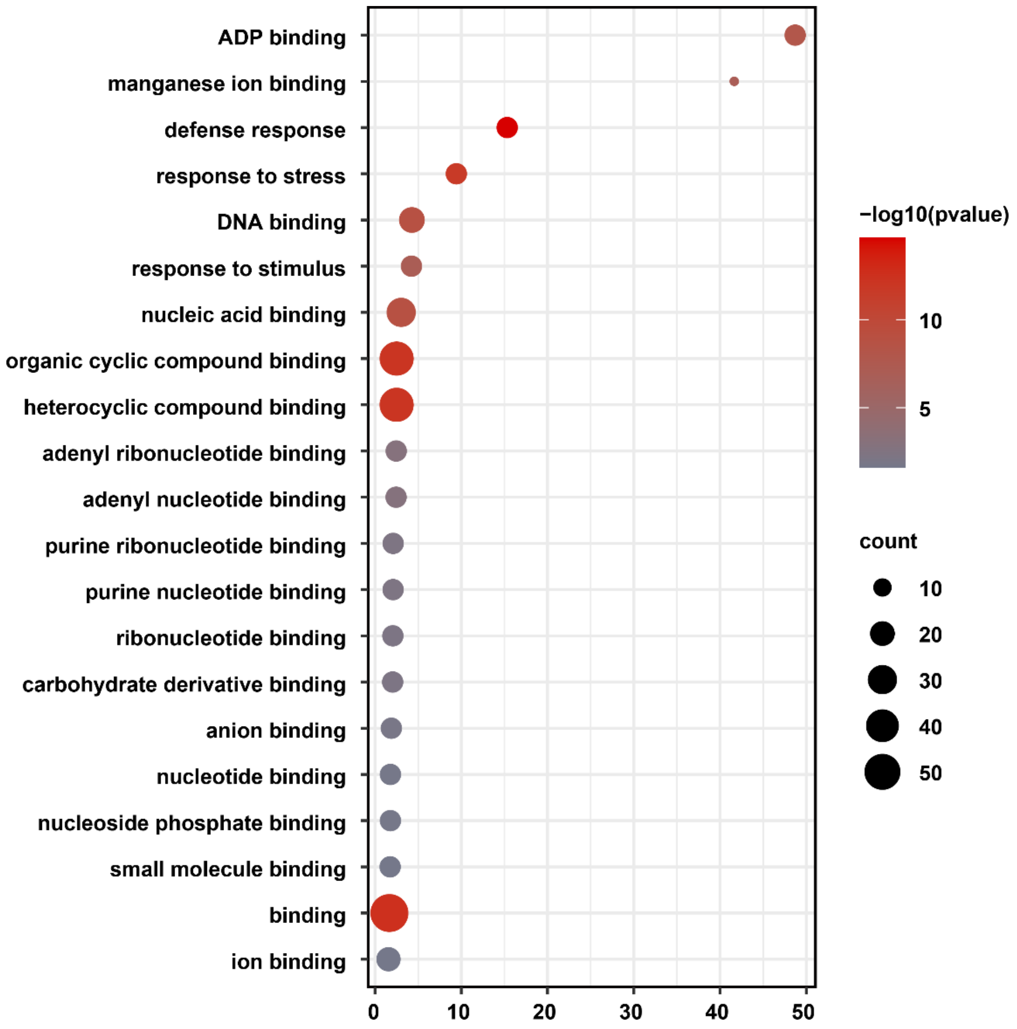

Supplementary Figure 16. The Gene Ontology (GO) enrichment analysis for genes with expansion in *M. caulescens*. Source data are provided as a Source Data file.

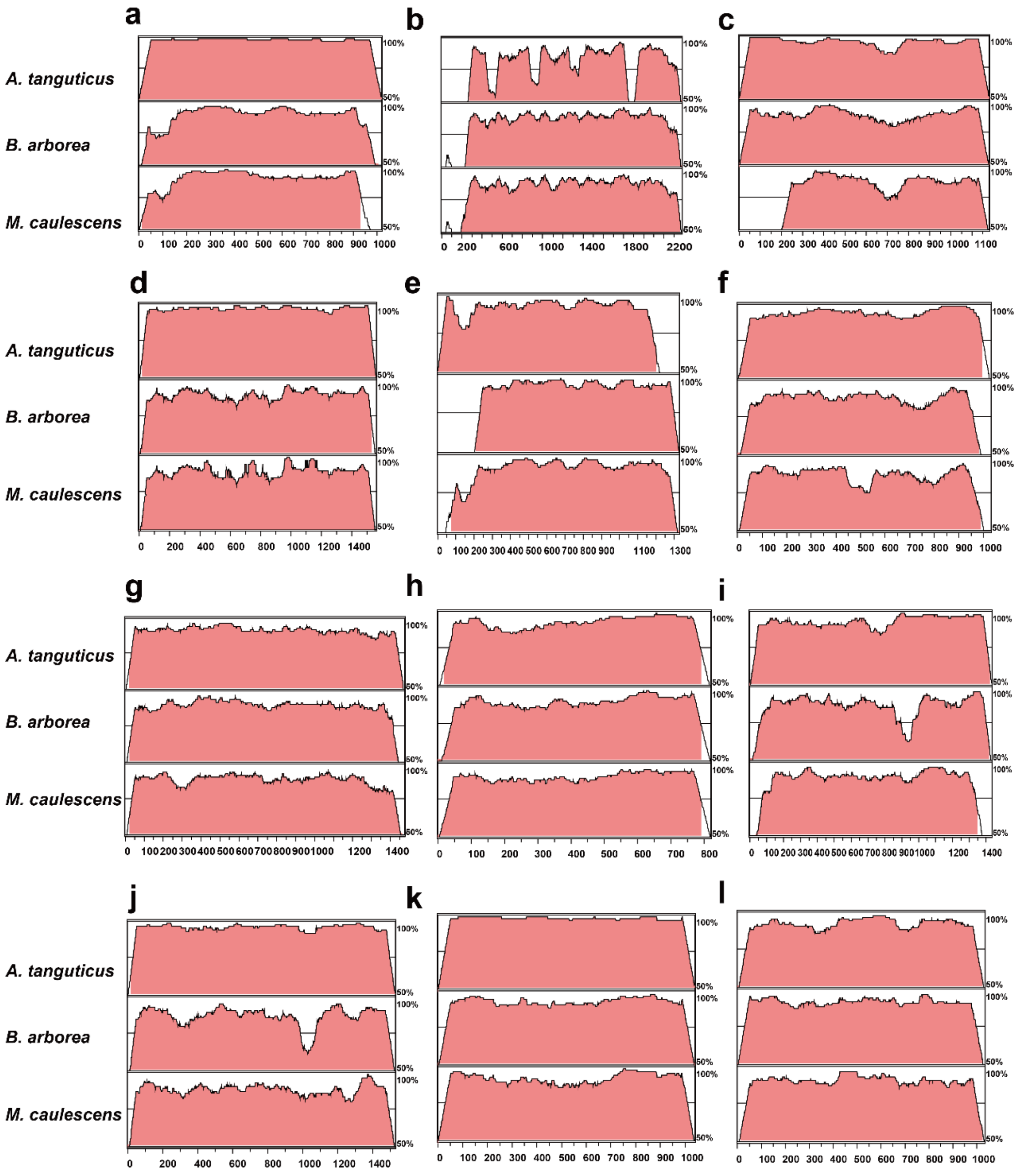

Supplementary Figure 17. VISTA sequence conservation plot between HS biosynthetic genes from three HS-producing species using *A. belladonna* as a reference. The genes analyzed in this figure including *PMT* (**a**), *MPO* (**b**), *PYKS* (**c**), *CYP82M3* (**d**), *AT4* (**e**), *PPAR* (**f**), *UGT1* (**g**), *TRI* (**h**), *LS* (**i**), *CYP80F1* (**j**), *HDH* (**k**) and *H6H* (l). The species used in this figure include *A. tanguticus, B. arborea* and *M. caulescens.*

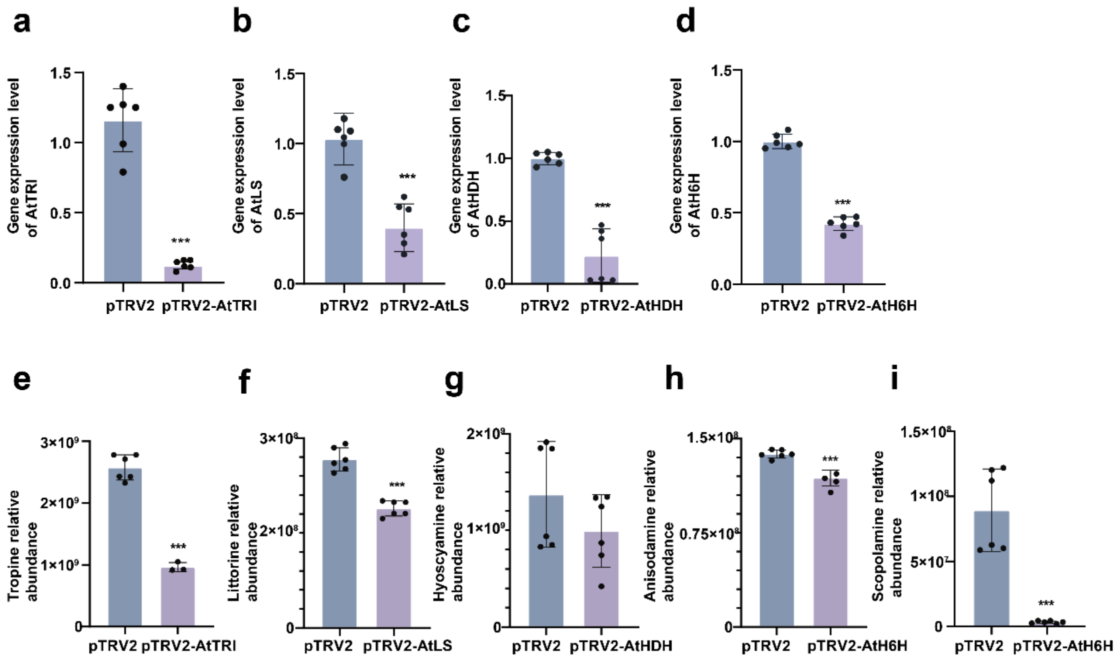

Supplementary Figure 18. The virus-induced gene silencing (VIGS) of *TRI*, *LS*, *HDH* and *H6H* in *A. tanguticus* seedlings. a-d, Data were obtained by quantitative RT-PCR and are presented as the mean ± SD with the expression level of *At*TRI (a), *At*LS (b), *At*HDH (c) and *At*H6H (d). pTRV2, control; pTRV2-*At*TRI, *TRI*-silenced plant; pTRV2-*At*LS, *LS*-silenced plant; pTRV2-*At*HDH, *HDH*-silenced plant; pTRV2-*At*H6H, *H6H*-silenced plant. Asterisks denote significant differences (*$p < 0.05$; **$p < 0.01$; ***$p < 0.001$) as determined by two-tailed Student's t test. For gene expression level, $p = 0.0001$ (*At*TRI), $p = 0.0001$ (*At*LS), $p = 0.0001$ (*At*HDH), $p = 0.0001$ (*At*H6H). e-I, The VIGS of *TRI*, *LS*, *HDH*, *H6H* in *A. tanguticus* seedlings affects tropane alkaloid biosynthesis. From e to i: the abundance of the tropine (e), littorine (f), hyoscyamine (g), anisodamine (h) and scopolamine (i). pTRV2, control; pTRV2-*At*TRI, *TRI*-silenced plant; pTRV2-*At*LS, *LS*-silenced plant; pTRV2-*At*HDH, *HDH*-silenced plant; pTRV2-*At*H6H, *H6H*-silenced plant. The data are presented as means values +/− s.d. Asterisks denote a significant difference (*$p < 0.05$; **$p < 0.01$; ***$p < 0.001$) as determined by two-tailed Student's t test. For alkaloids contents, $p = 0.0001$ (tropine), $p = 0.0001$ (littorine), $p = 0.1944$ (hyoscyamine), $p = 0.0002$ (anisodamine), $p = 0.0001$ (scopolamine). Source data are provided as a Source Data file.

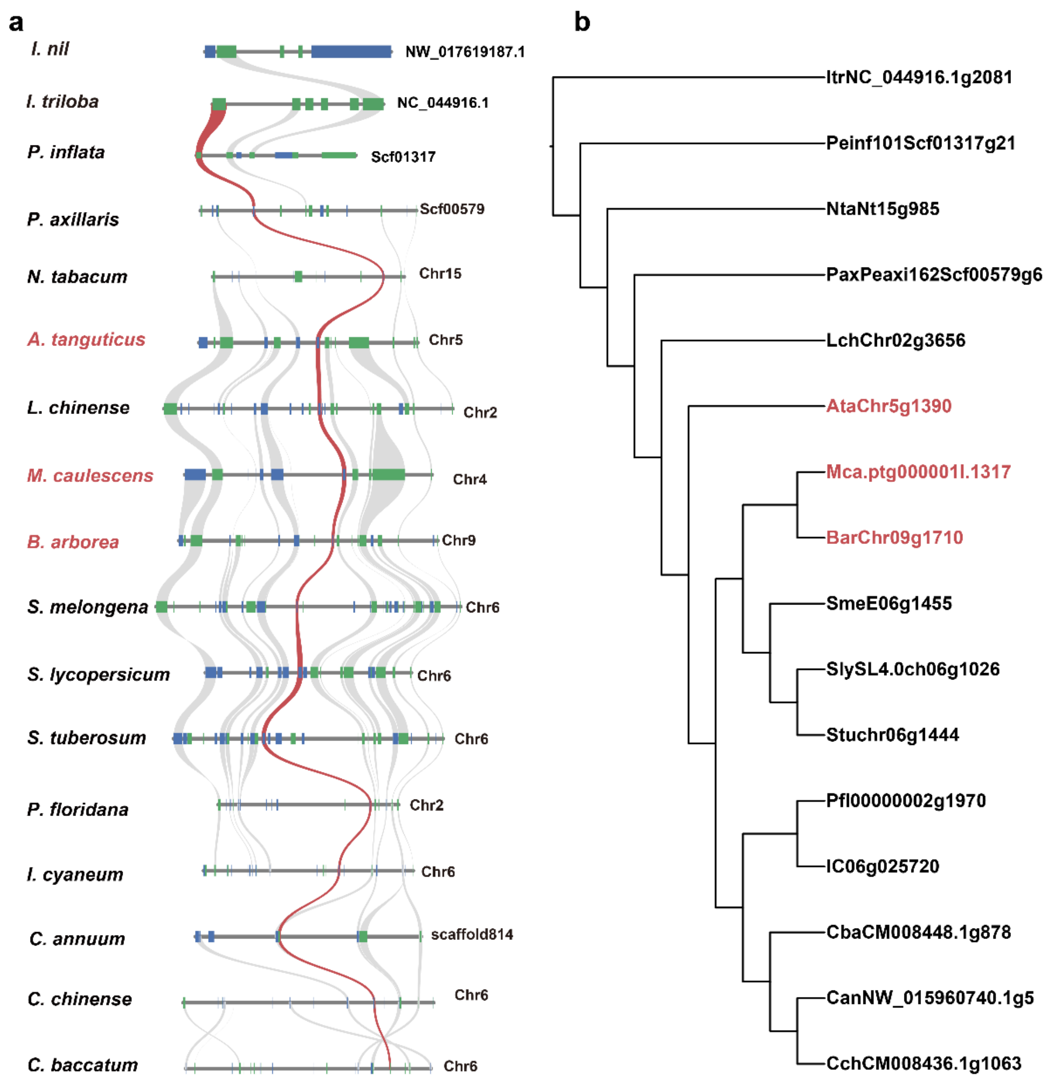

Supplementary Figure 19. The collinearity analysis of *PMT* regions in 15 Solanaceae genomes and phylogeny. a, The collinearity analysis of *PMT* regions. Rectangles represent annotated genes with orientation on the reverse strand (green) and same strand (blue). The lines link the syntenic *PMT* genes are highlighted in red. The grey lines represent the gene collinearity among candidate species. b, Gene trees constructed from *PMT* collinearity genes with *I. nil* and *I. triloba* of Convolvulaceae as outgroup. Shown are here genes names represented as species abbreviations and genes. Ata: *Anisodus tanguticus*; Bar: *Brugmansia arborea*; Mca: *Mandragora caulescens*; Can: *Capsicum annuum*; Cba: *Capsicum baccatum*; Cch: *Capsicum chinense*; Lch: *Lycium chinense*; Nta: *Nicotiana tabacum*; Pfl: *Physalis floridana*; Sly: *Solanum lycopersicum*; Sme: *Solanum melongena*; Stu: *Solanum tuberosum*; Pax: *Petunia axillaris*, IC: *Iochroma cyaneum*. Pin: *P. inflata*; Ini: *I. nil*; Itr: *I. triloba*. Red highlights denote HS-producing species.

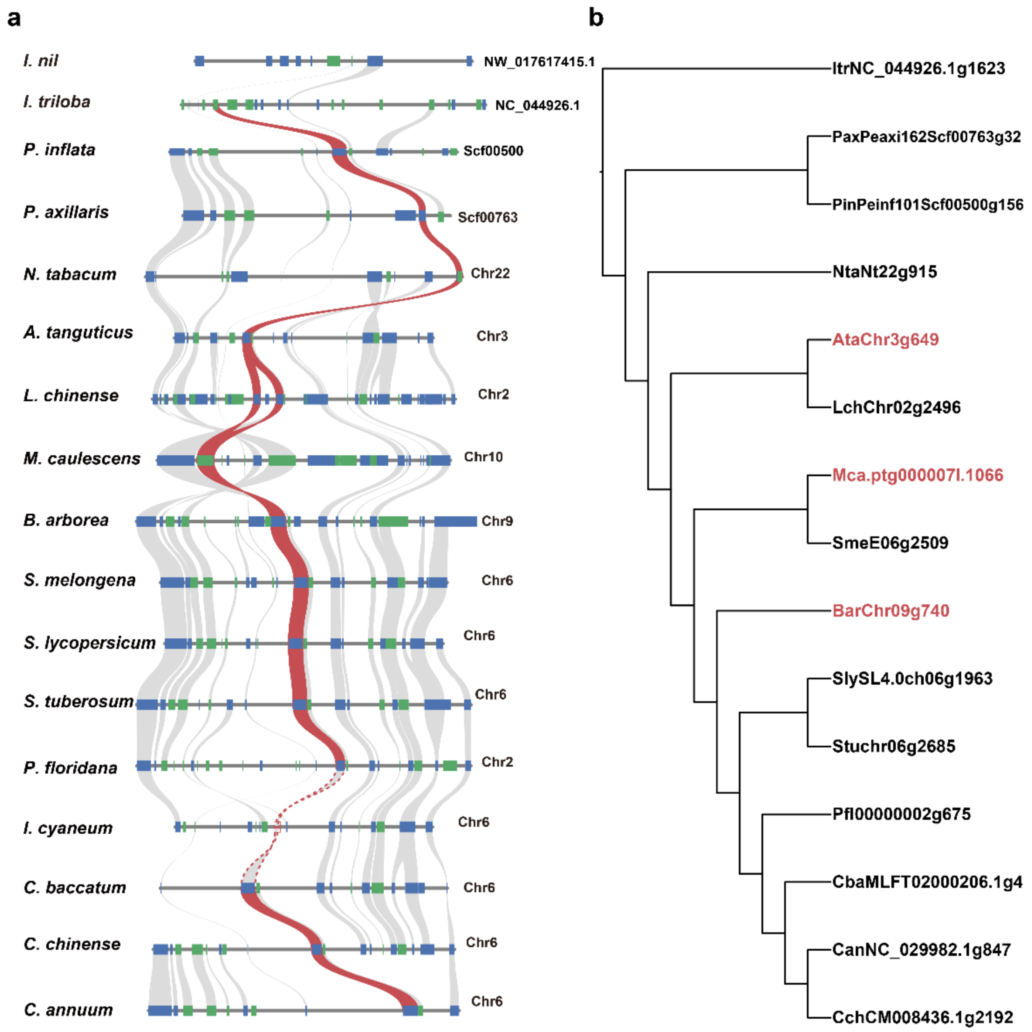

Supplementary Figure 20. The collinearity analysis of *MPO* regions in 15 Solanaceae genomes and phylogeny. a, The collinearity analysis of *MPO* regions. Rectangles represent annotated genes with orientation on the reverse strand (green) and same strand (blue). *MPO* gene names of *A. tanguticus, B. arborea* and *M. caulescens* were marked above the gene blocks. The lines link the syntenic *MPO* genes are highlighted in red. The grey lines represent the gene collinearity among candidate species. b, Gene trees constructed from *MPO* collinearity genes with *I. nil* and *I. triloba* of Convolvulaceae as outgroup. Shown are here genes names represented as species abbreviations and genes. Ata: *Anisodus tanguticus*; Bar: *Brugmansia arborea*; Mca: *Mandragora caulescens*; Can: *Capsicum annuum*; Cba: *Capsicum baccatum*; Cch: *Capsicum chinense*; Lch: *Lycium chinense*; Nta: *Nicotiana tabacum*; Pfl: *Physalis floridana*; Sly: *Solanum lycopersicum*; Sme: *Solanum melongena*; Stu: *Solanum tuberosum*; Pax: *Petunia axillaris*; Ini: *I. nil*; Itr: *I. trilobal*; IC: *Iochroma cyaneum* Red highlights denote HS-producing species.

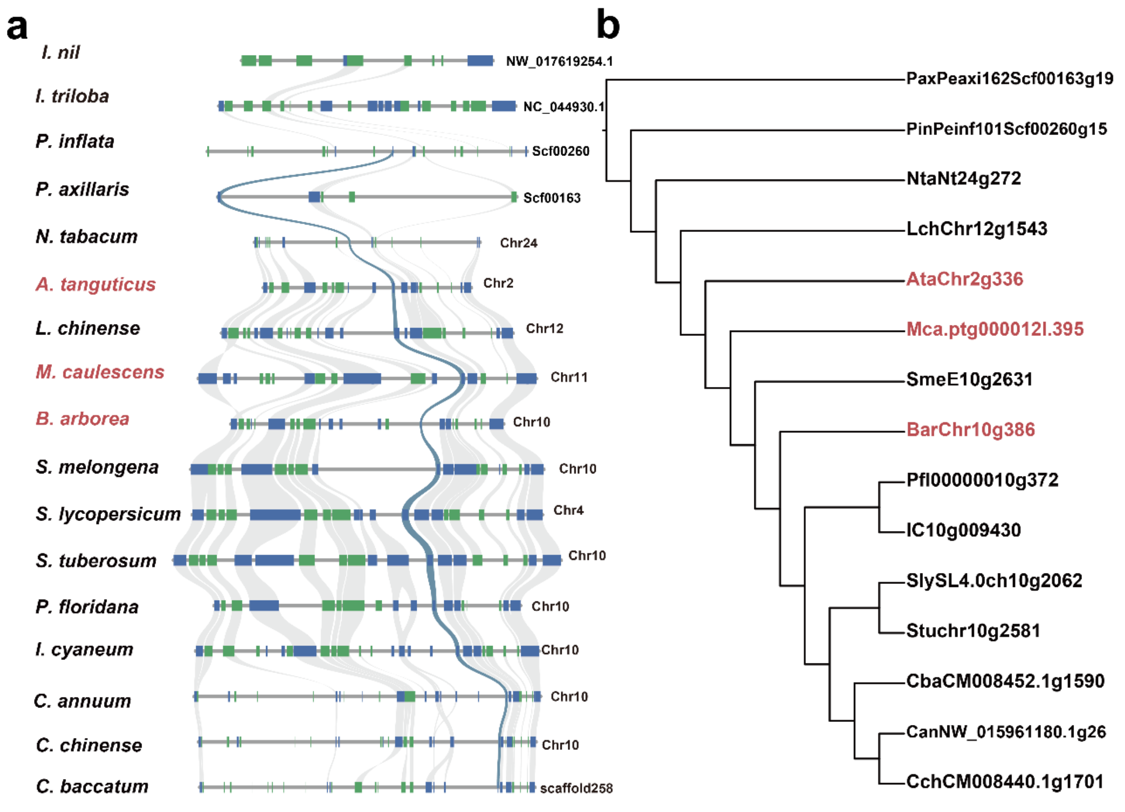

Supplementary Figure 21. The collinearity analysis of *CYP82M3* regions in 15 Solanaceae genomes and phylogeny. a, The collinearity analysis of *CYP82M3* regions. Rectangles represent annotated genes with orientation on the reverse strand (green) and same strand (blue). *CYP82M3* gene names of *A. tanguticus, B. arborea* and *M. caulescens* were marked above the gene blocks. The lines link the syntenic *CYP82M3* genes are highlighted in blue. The grey lines represent the gene collinearity among candidate species. b, Gene trees constructed from *CYP82M3* collinearity genes with *P. inflata* and *P. axillaris* of scaffold level genome as outgroup. Shown are here genes names represented as species abbreviations and genes. Ata: *Anisodus tanguticus*; Bar: *Brugmansia arborea*; Mca: *Mandragora caulescens*; Can: *Capsicum annuum*; Cba: *Capsicum baccatum*; Cch: *Capsicum chinense*; Lch: *Lycium chinense*; Nta: *Nicotiana tabacum*; Pfl: *Physalis floridana*; Sly: *Solanum lycopersicum*; Sme: *Solanum melongena*; Stu: *Solanum tuberosum*; Pax: *Petunia axillaris*, Pin: *P. inflata;* IC: *Iochroma cyaneum.* Red highlights denote HS-producing species.

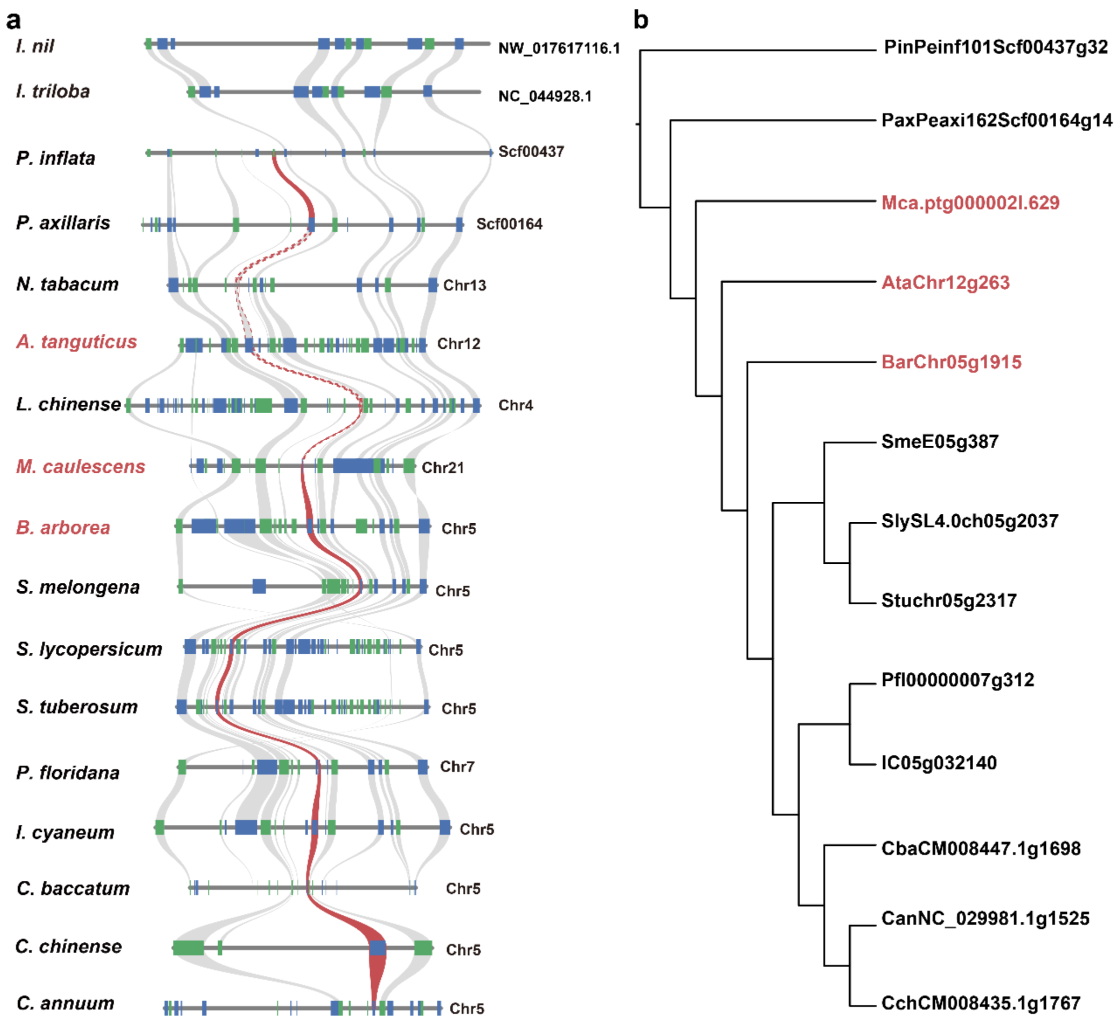

Supplementary Figure 22. The collinearity analysis of *PYKS* regions in 15 Solanaceae genomes and phylogeny. a, The collinearity analysis of *PYKS* regions. Rectangles represent annotated genes with orientation on the reverse strand (green) and same strand (blue). *PYKS* gene names of *A. tanguticus, B. arborea* and *M. caulescens*; were marked above the gene blocks. The lines link the syntenic *PYKS* genes are highlighted in red. The grey lines represent the gene collinearity among candidate species. b, Gene trees constructed from *PYKS* collinearity genes with *P. inflata* of scaffold level genome as outgroup. Shown are here genes names represented as species abbreviations and genes. Ata: *Anisodus tanguticus*; Bar: *Brugmansia arborea*; Mca: *Mandragora caulescens*; Can: *Capsicum annuum*; Cba: *Capsicum baccatum*; Cch: *Capsicum chinense*; Pfl: *Physalis floridana*; Sly: *Solanum lycopersicum*; Sme: *Solanum melongena*; Stu: *Solanum tuberosum*; Pax: *Petunia axillaris*, Pin: *P. inflata*; IC: *Iochroma cyaneum.* Red highlights denote HS-producing species.

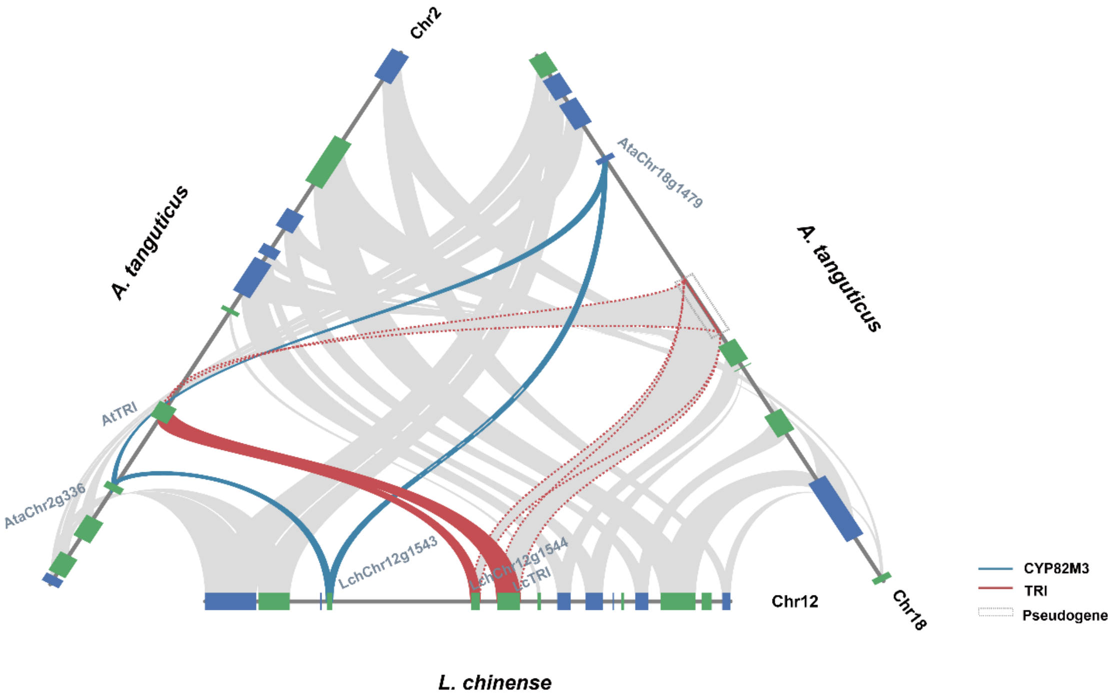

Supplementary Figure 23. The synteny of *TRI* and *CYP82M3* between *A. tanguticus* and *L. chinense*. Rectangles represent annotated genes with orientation on the reverse strand (green) and same strand (blue). *CYP82M3* and *TRI* gene names of *A. tanguticus* and *L. chinense* were marked above the gene blocks. The lines link the syntenic *TRI* genes are highlighted in red and *CYP82M3* genes are highlighted in blue. The red dashed represent assumed collinearity of incomplete *TRI* genes (pseudogene) from *A. tanguticus*. The grey lines represent the gene collinearity among candidate species.

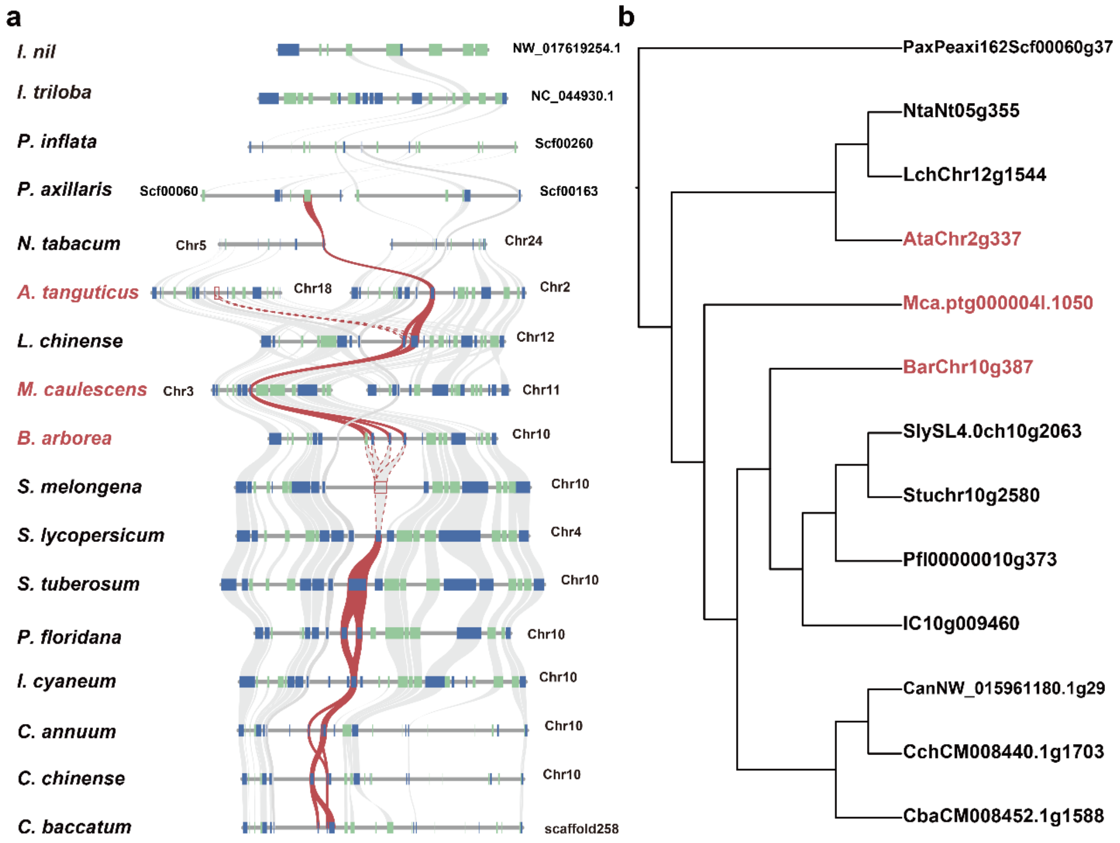

Supplementary Figure 24. The collinearity analysis of *TRI* regions in 15 Solanaceae genomes and phylogeny. a, The collinearity analysis of *TRI* regions. Rectangles represent annotated genes with orientation on the reverse strand (green) and same strand (blue). *TRI* gene names of *A. tanguticus, B. arborea* and *M. caulescens* were marked above the gene blocks. The lines link the syntenic *TRI* genes are highlighted in red. The grey lines represent the gene collinearity among candidate species. b, Gene trees constructed from *TRI* collinearity genes with *P. axillaris* of scaffold level genome as outgroup. Shown are here genes names represented as species abbreviations and genes. Ata: *Anisodus tanguticus*; Bar: *Brugmansia arborea*; Mca: *Mandragora caulescens*; Can: *Capsicum annuum*; Cba: *Capsicum baccatum*; Cch: *Capsicum chinense*; Lch: *Lycium chinense*; Pfl: *Physalis floridana*; Sly: *Solanum lycopersicum*; Stu: *Solanum tuberosum*; Pax: *Petunia axillaris*, IC: *Iochroma cyaneum.* Red highlights denote HS -producing species.

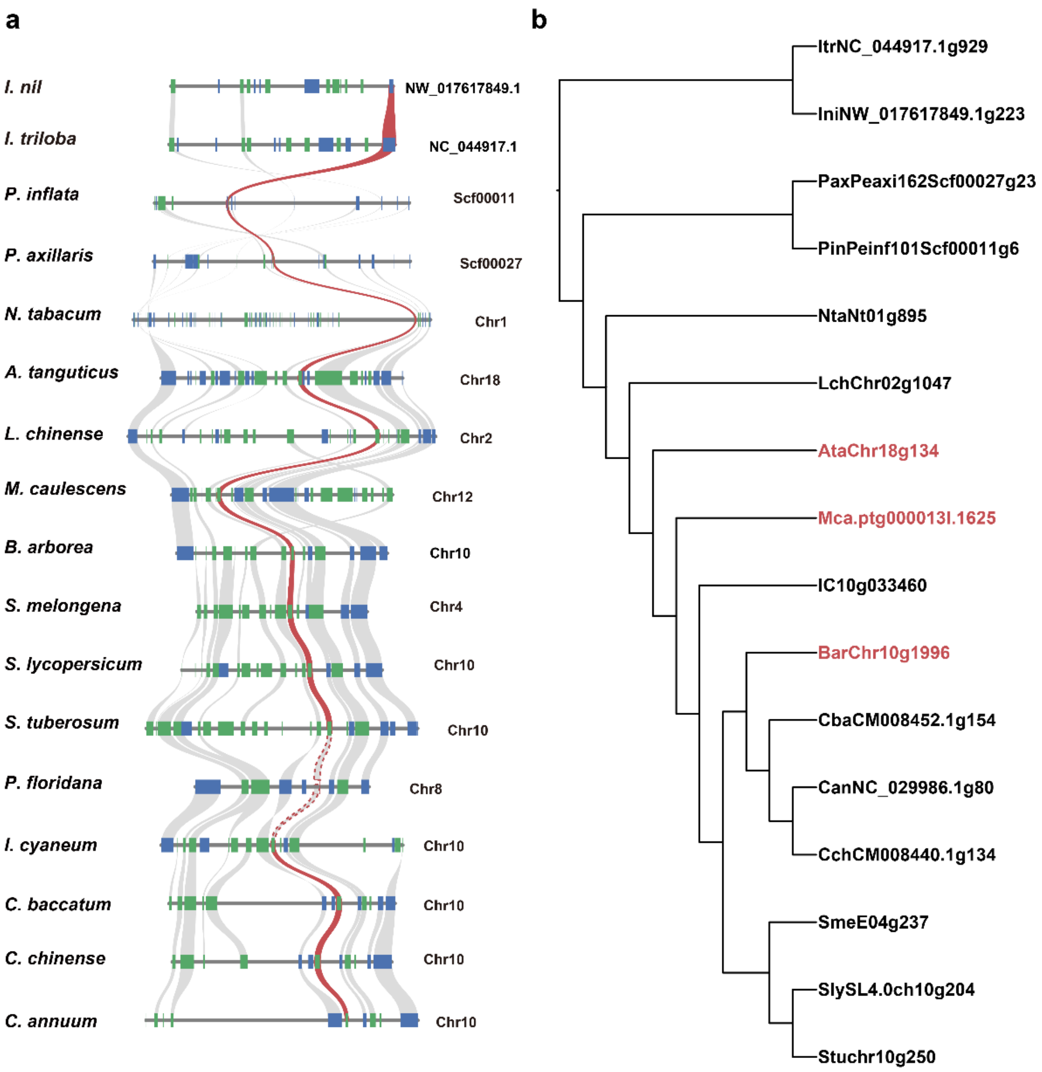

Supplementary Figure 25. The collinearity analysis of *AT4* regions in 15 Solanaceae genomes and phylogeny. a, Rectangles represent annotated genes with orientation on the reverse strand (green) and same strand (blue). *AT4* gene names of *A. tanguticus, B. arborea* and *M. caulescens* were marked above the gene blocks. The lines link the syntenic *AT4* genes are highlighted in red. The grey lines represent the gene collinearity among candidate species. b, Gene trees constructed from *AT4* collinearity genes with *I. nil* and *I. triloba* of Convolvulaceae as outgroup. Shown are here genes names represented as species abbreviations and genes. Ata: *Anisodus tanguticus*; Bar: *Brugmansia arborea*; Mca: *Mandragora caulescens*; Can: *Capsicum annuum*; Cba: *Capsicum baccatum*; Cch: *Capsicum chinense*; Lch: *Lycium chinense*; Pfl: *Physalis floridana*; Sly: *Solanum lycopersicum*; Sme: *Solanum melongena*; Stu: *Solanum tuberosum*, Pax: *Petunia axillaris*, Pin: *P. inflata*; *Ini: I. nil*; *Itr: I. trilobal*; IC: *Iochroma cyaneum.* Red highlights denote HS-producing species.

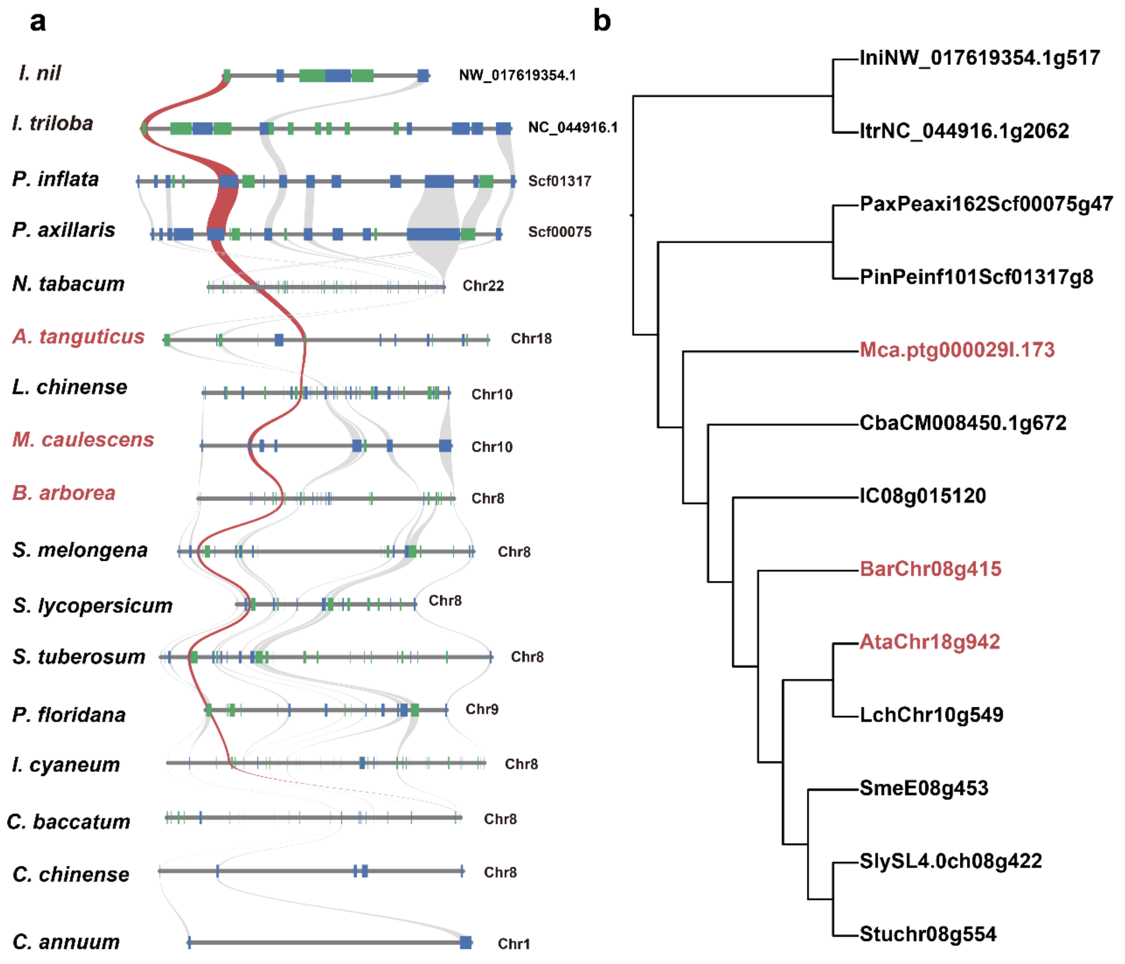

Supplementary Figure 26. The collinearity analysis of *UGT1* regions in 15 Solanaceae genomes and phylogeny. a, The collinearity analysis of *UGT1* regions. Rectangles represent annotated genes with orientation on the reverse strand (green) and same strand (blue). *UGT1* gene names of *A. tanguticus, B. arborea* and *M. caulescens* were marked above the gene blocks. The lines link the syntenic *UGT1* genes are highlighted in red. The grey lines represent the gene collinearity among candidate species. b, Gene trees constructed from *UGT1* collinearity genes with *I. nil* and *I. triloba* of Convolvulaceae as outgroup. Shown are here genes names represented as species abbreviations and genes. Ata: *Anisodus tanguticus*; Bar: *Brugmansia arborea*; Mca: *Mandragora caulescens*; Cba: *Capsicum baccatum*; Lch: *Lycium chinense*; Sly: *Solanum lycopersicum*; Sme: *Solanum melongena*; Stu: *Solanum tuberosum*; Pax: *Petunia axillaris*, Pin: *inflata,* IC: *Iochroma cyaneum*; *Ini*: *I. nil*; *Itr: I. trilobal*; IC: *Iochroma cyaneum.* Red highlights denote HS-producing species.

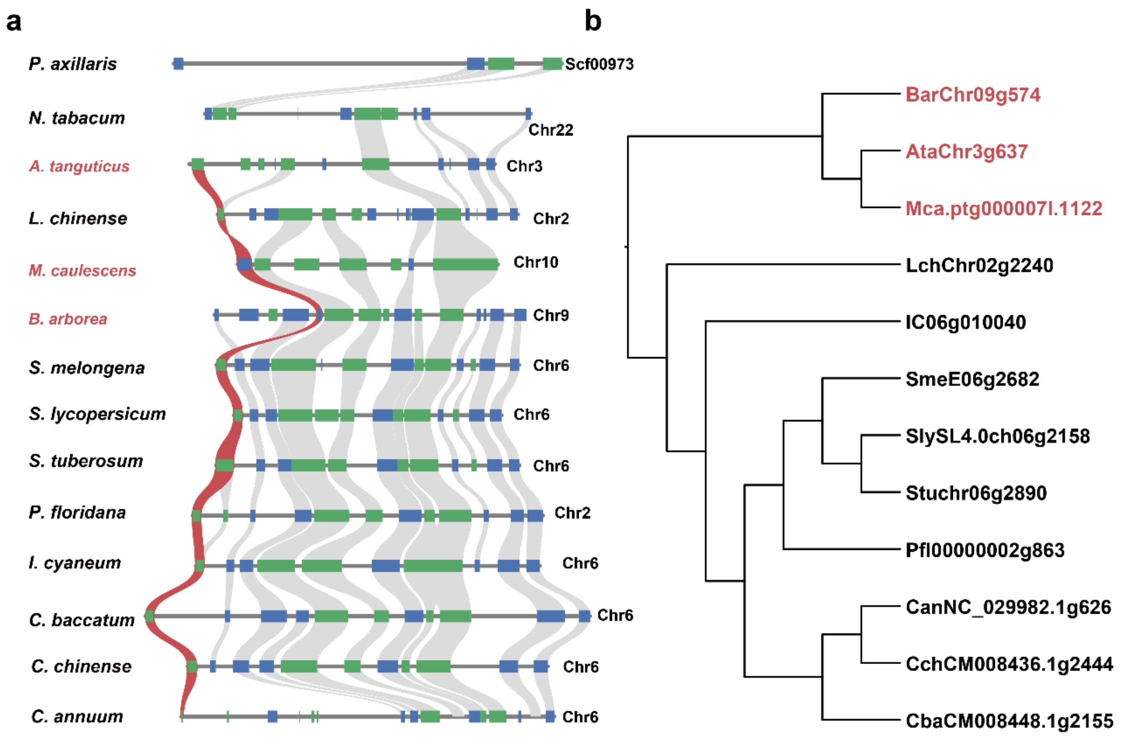

Supplementary Figure 27. The collinearity analysis of *H6H* regions in 15 Solanaceae genomes and phylogeny. a, Rectangles represent annotated genes with orientation on the reverse strand (green) and same strand (blue). *H6H* gene names of *A. tanguticus, B. arborea* and *M. caulescens* were marked above the gene blocks. The lines link the syntenic *H6H* genes are highlighted in red. The grey lines represent the gene collinearity among candidate species. b, Gene trees constructed from *H6H* collinearity genes. Shown are here genes names represented as species abbreviations and genes. Ata: *Anisodus tanguticus*; Bar: *Brugmansia arborea*; Mca: *Mandragora caulescens*; Can: *Capsicum annuum*; Cba: *Capsicum baccatum*; Cch: *Capsicum chinense*; Lch: *Lycium chinense*; Pfl: *Physalis floridana*; Sly: *Solanum lycopersicum*; Sme: *Solanum melongena*; Stu: *Solanum tuberosum*; Ioc: *Iochroma cyaneum*. Red highlights denote HS-producing species.

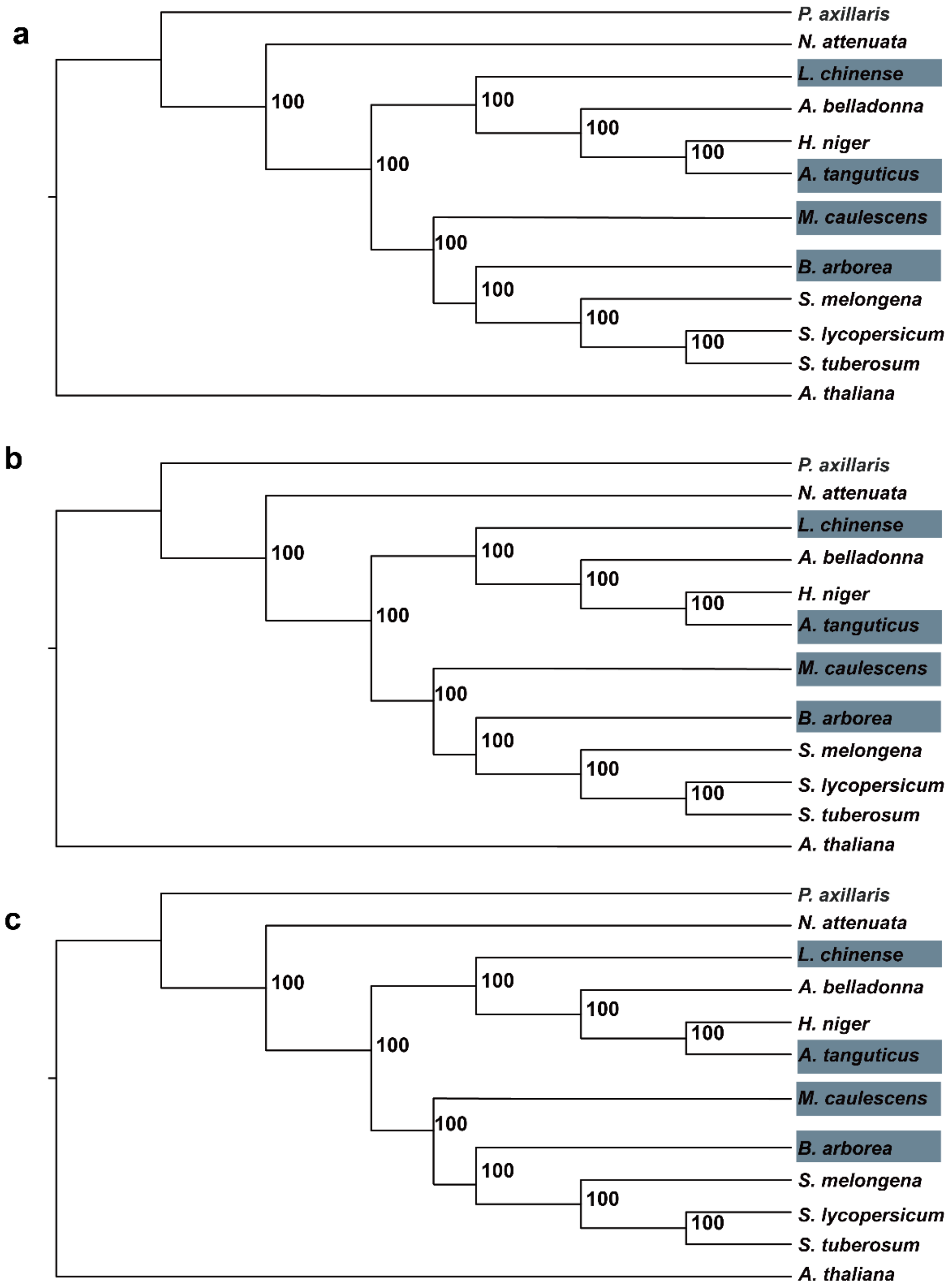

Supplementary Figure 28. A The phylogenetic tree between the sampled species of the Solanaceae based on phylogenetic analyses of three data sets (chloroplast genomes, single-copy nuclear genes and BUSCO conserved genes). a, chloroplast genomes dataset. b, single-copy nuclear genes dataset. A total of 256 single-copy genes are used to construct species tree. c, BUSCO conserved genes dataset. A total of 257 BUSCO conserved genes are used to construct species tree. Source data are provided as a Source Data file.

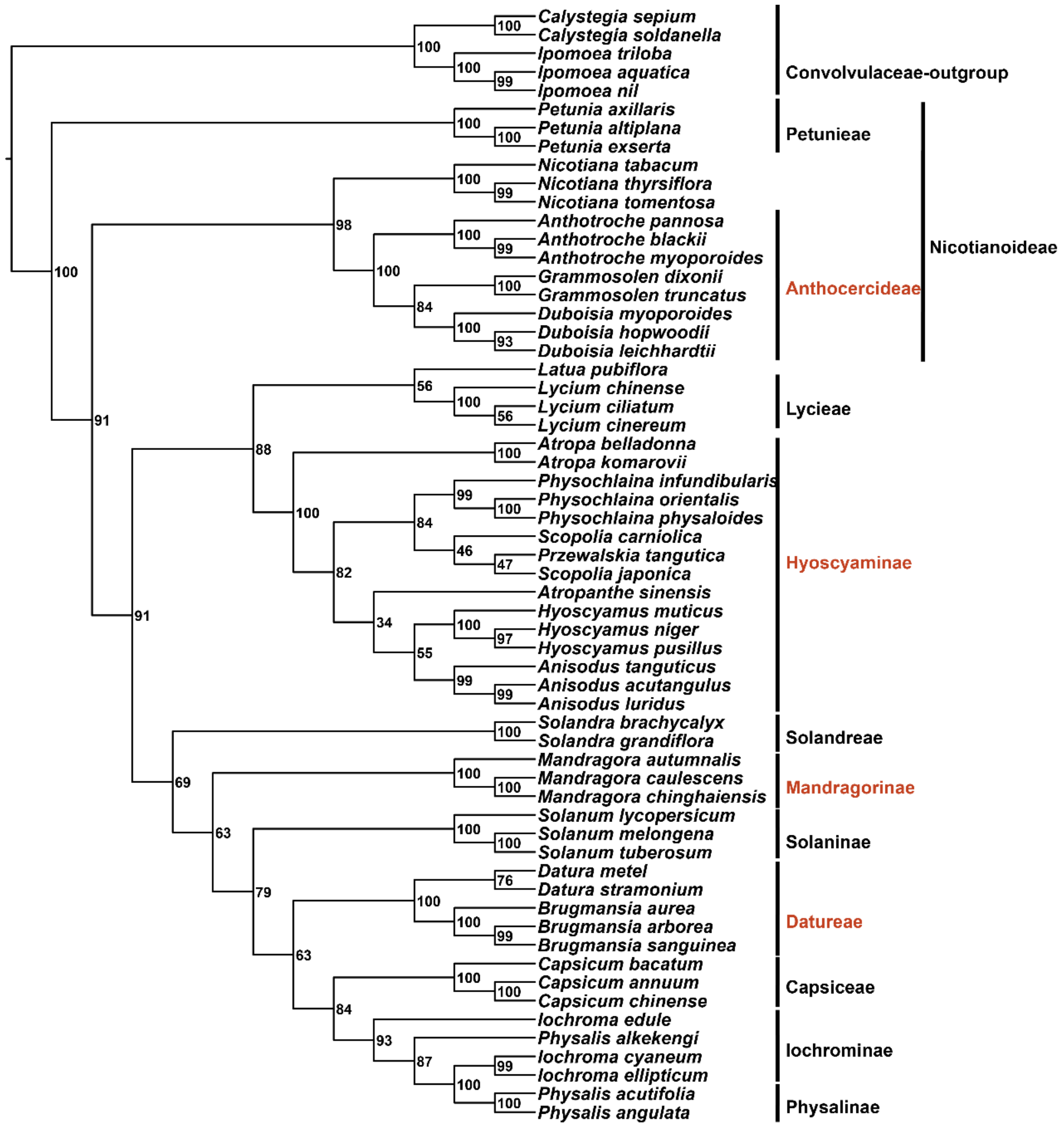

Supplementary Figure 29. The phylogenetic tree of Solanaceae based on *ndhF*, *matK*, trans/G, trnL/F concatenation sequences using Convolvulaceae species as outgroups. Red highlights denote HS-producing species from four tribes in Solanaceae. Source data are provided as a Source Data file.

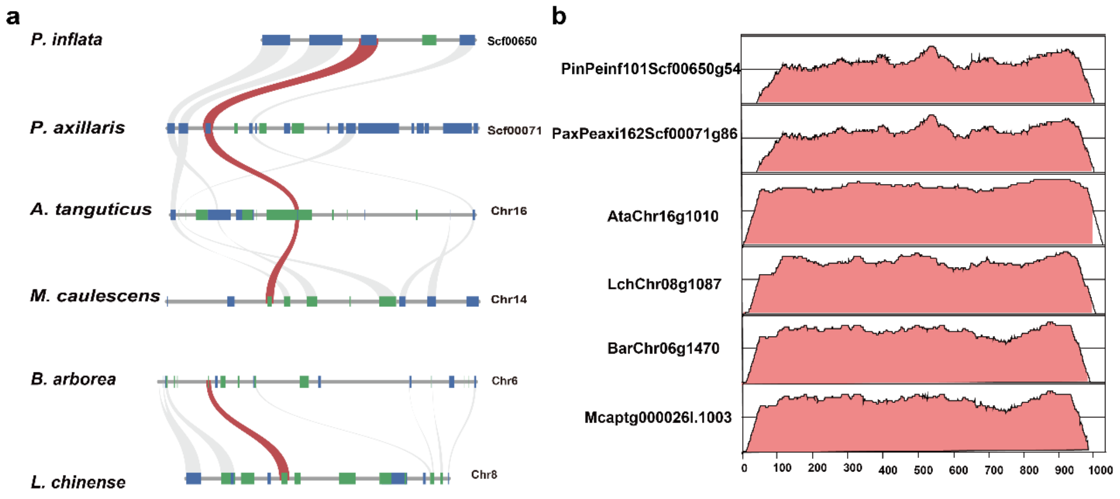

Supplementary Figure 30. The synteny and sequences similarity of *PPAR* gene. a, The synteny of *PPAR* between *P. inflata, P. axillaris*, *A. tanguticus*, and *M. caulescens* (up). The synteny of *PPAR* between *B. arborea* and *L. chinense* (down). Rectangles represent annotated genes with orientation on the reverse strand (green) and same strand (blue). The lines link the syntenic *PPAR* genes are highlighted in red. The grey lines represent the gene collinearity among candidate species. b, VISTA sequence conservation plot of the *PPAR* gene using *A. belladonna* as a reference. Ata: *Anisodus tanguticus*; Bar: *Brugmansia arborea*; Mca: *Mandragora caulescens*; Lch: *Lycium chinense*; Pax: *Petunia axillaris*, Pin: *P. inflata.*

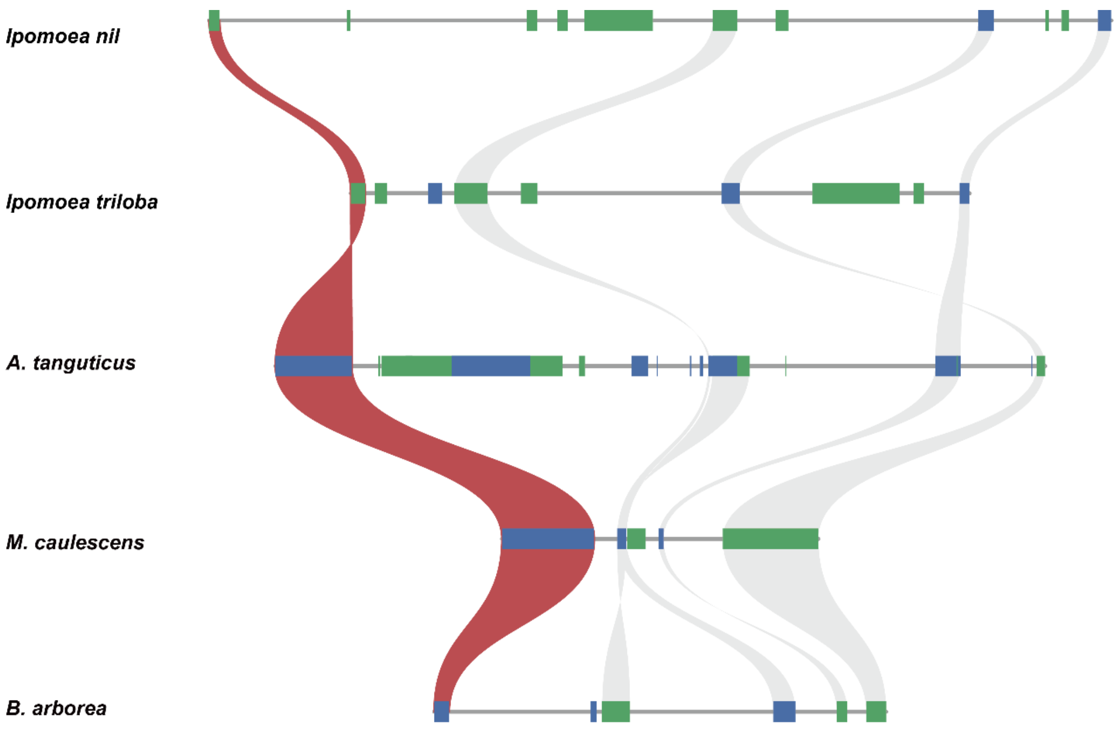

Supplementary Figure 31. The collinearity analysis of *HDH* regions in three HS-producing species (*A. tanguticus*, *M. caulescens* and *B. arborea*) genomes and *Ipomoea triloba*, *Ipomoea nil*. Rectangles represent annotated genes with orientation on the reverse strand (green) and same strand (blue). The lines link the syntenic *HDH* genes are highlighted in red. The grey lines represent the gene collinearity among candidate species.

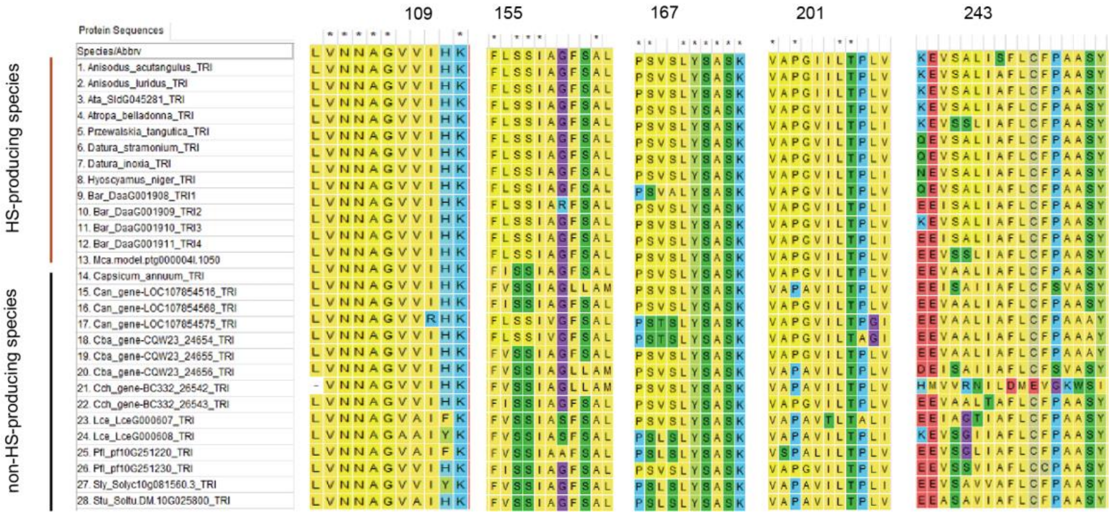

Supplementary Figure 32. Amino acid sequences of TRI alignment between 17 Solanaceae species. Shown are here genes names represented as species abbreviations and genes. Ata: *Anisodus tanguticus*; Bar: *Brugmansia arborea*; Can: *Capsicum annuum*; Cba: *Capsicum baccatum*; Cch: *Capsicum chinense*; Lce: *Lycium chinense*; Pfl: *Physalis floridana*; Sly: *Solanum lycopersicum*; Stu: *Solanum tuberosum*; Mca: *M. caulescens*; *Anisodus acutangulus*_TRI: EU424321.1; *Anisodus luridus*_TRI: KC713800.1; *Atropa belladonna*_TRI: JX155757.1; *Przewalskia tangutica*_TRI: MH165275.1; *Datura stramonium*_TRI: L20473.1; *Datura inoxia*_TRI: KJ676865.1; *Hyoscyamus niger*_TRI: D88156.1.

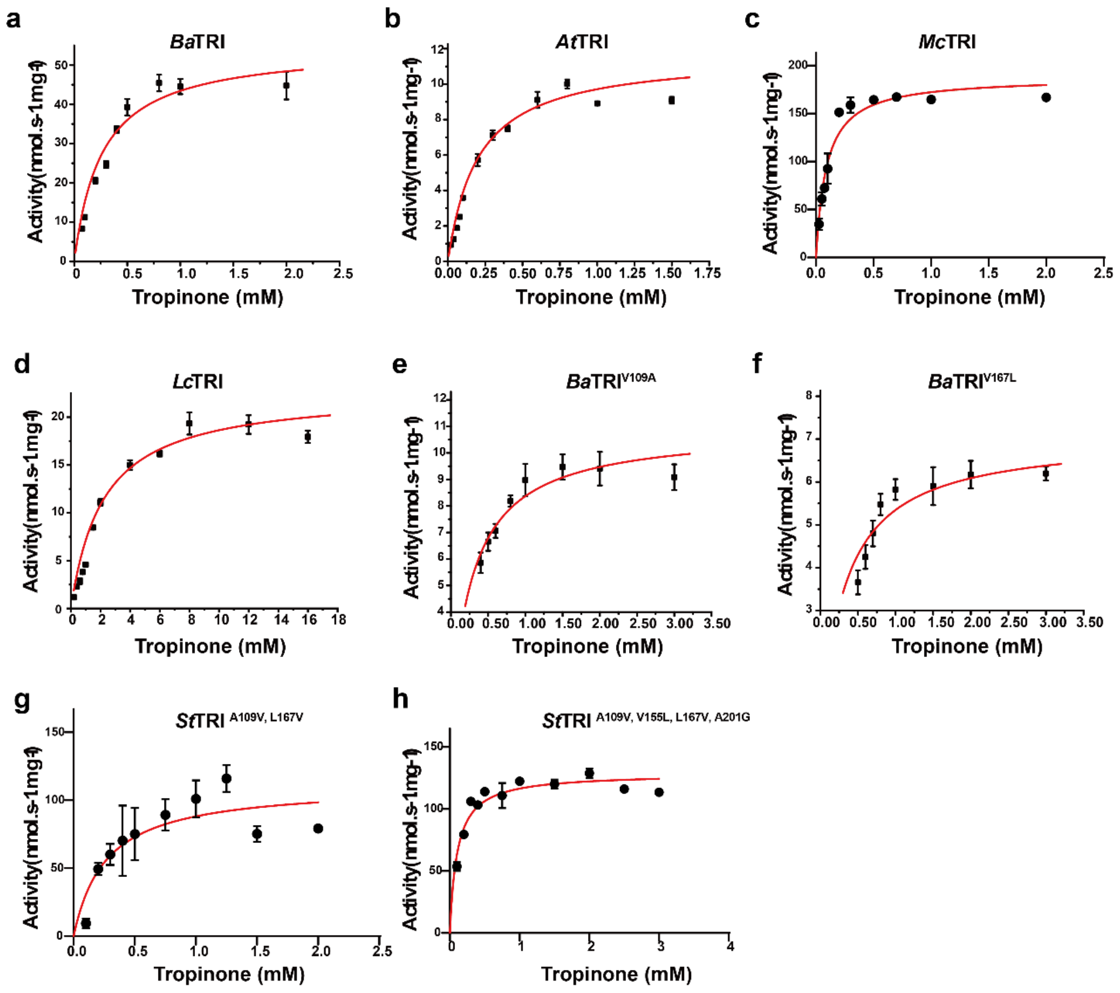

Supplementary Figure 33. Michaelis-Menten curves for the NADPH-dependent reduction of tropinone. a, Determination of Michaelis-Menten enzyme kinetic parameters of *Ba*TRI. b, *At*TRI. c, *Mc*TRI. d, *Lc*TRI. e, *Ba*TRI^{V109A}. f, *Ba*TRI^{V167L}. g, *St*TRI $^{A109V,\ L167V}$. h, *St*TRI $^{A109V,\ V155L,\ L167V,\ A201G}$. Each point is the mean from triplicate assays. Source data are provided as a Source Data file.

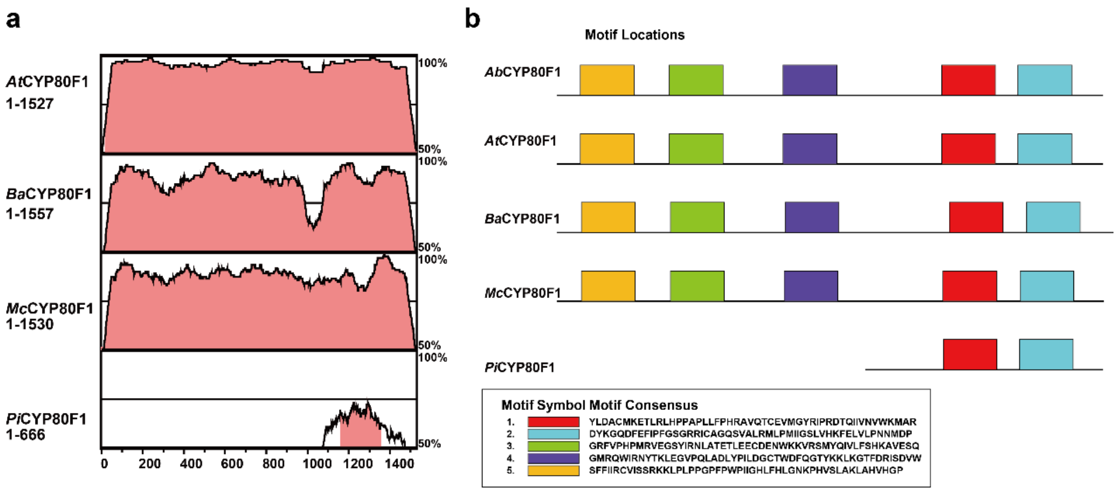

Supplementary Figure 34. Comparison of *CYP80F1* genes from *P. inflata* and other HS-producing species. a, VISTA sequence conservation plot between *CYP80F1* gene from *P. inflata* and other HS-producing species using *A. belladonna* as a reference. b, The motif of *P. inflata* and other HS-producing species.

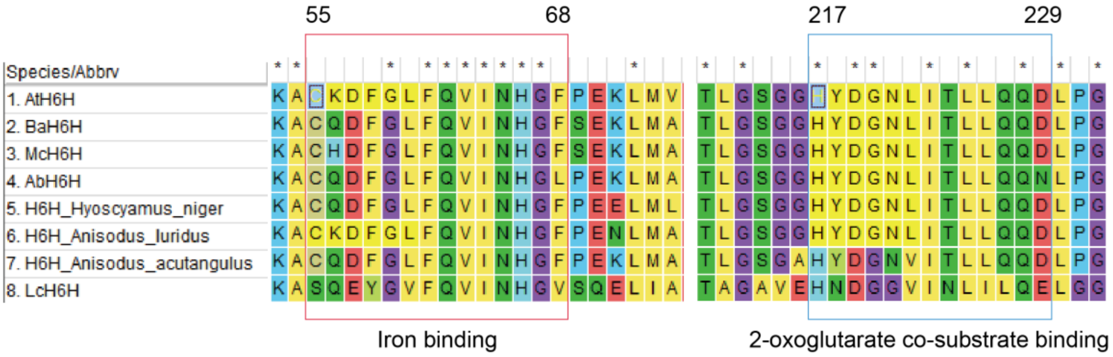

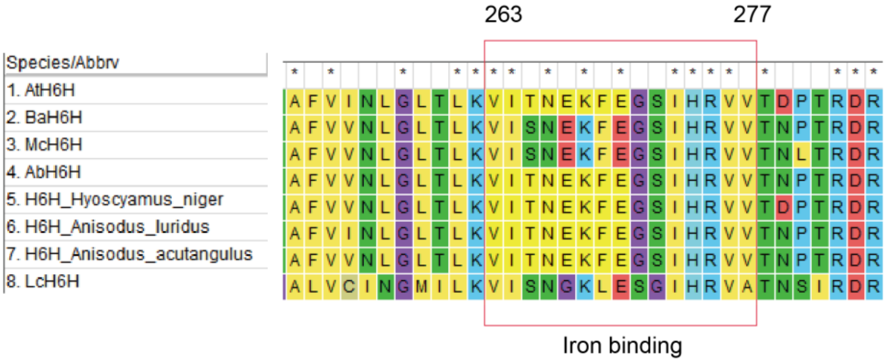

Supplementary Figure 35. Amino acid sequences alignment of *Lc*H6H and representative hyoscyamine 6β-hydroxylase from other solanaceous plants. Shown are here genes names represented as species abbreviations and genes. At: *Anisodus tanguticus*; Ba: *Brugmansia arborea*; Mc: *M. caulescens*; Ab: *A. belladonna*; Lc: *L. chinense.* H6H_*Hyoscyamus niger*: AAA33387.1; H6H_*Anisodus luridus*: AGL76991.1; H6H_*Anisodus acutangulus*: ABM74185.1. The two iron binding regions are in the red boxes, and the 2-oxoglutrate co-substrate binding site is in the blue box.

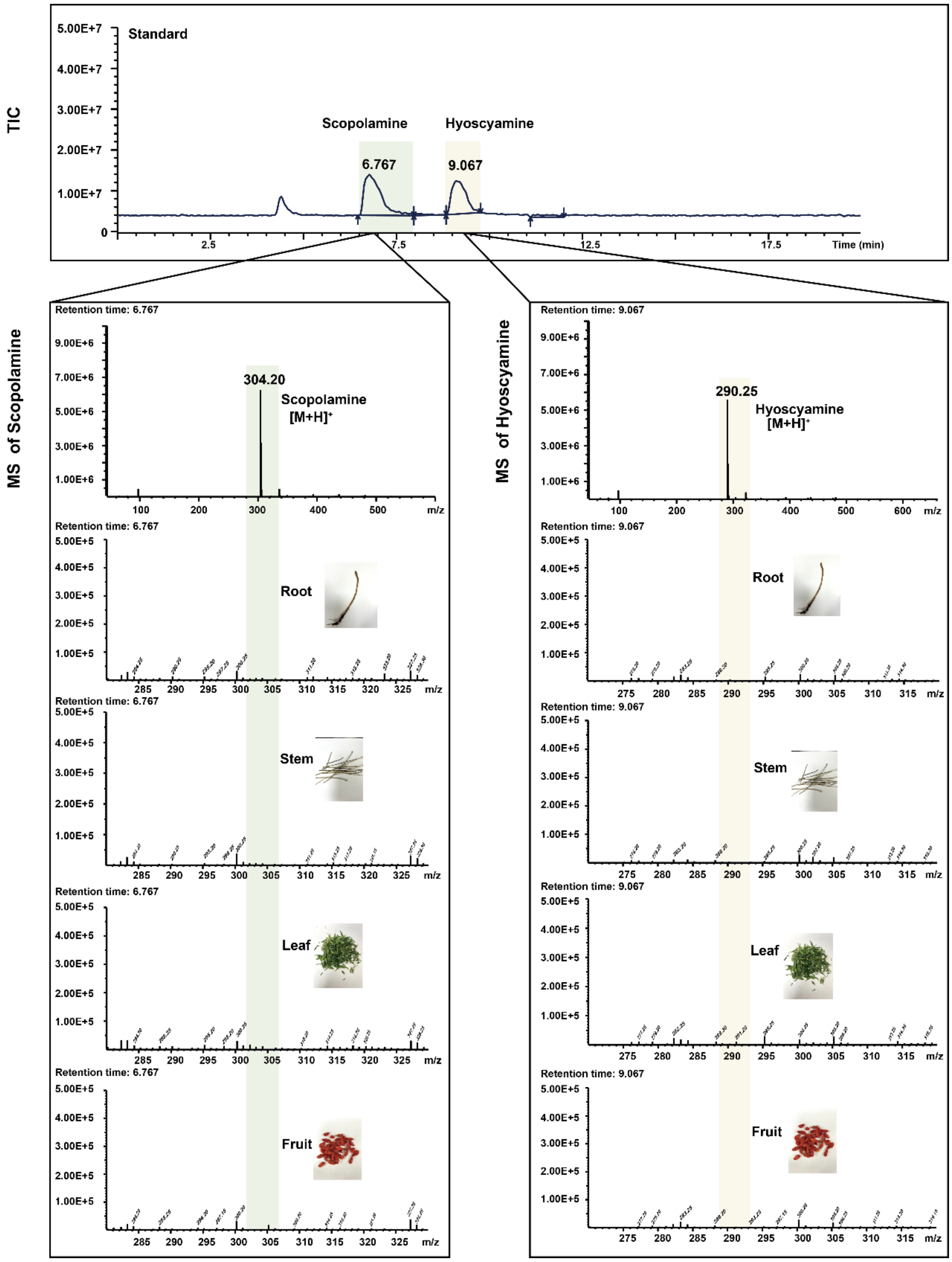

Supplementary Figure 36. MS spectra for determination of hyoscyamine and scopolamine in various tissues of *L. chinense*. TIC: Total Ion Chromatography. For MS model, the ions scopolamine (m/z, 304.20) and hyoscyamine (m/z, 290.25) was scanned between 285-325 and 275-315 in various tissues of *L. chinense*, respectively.

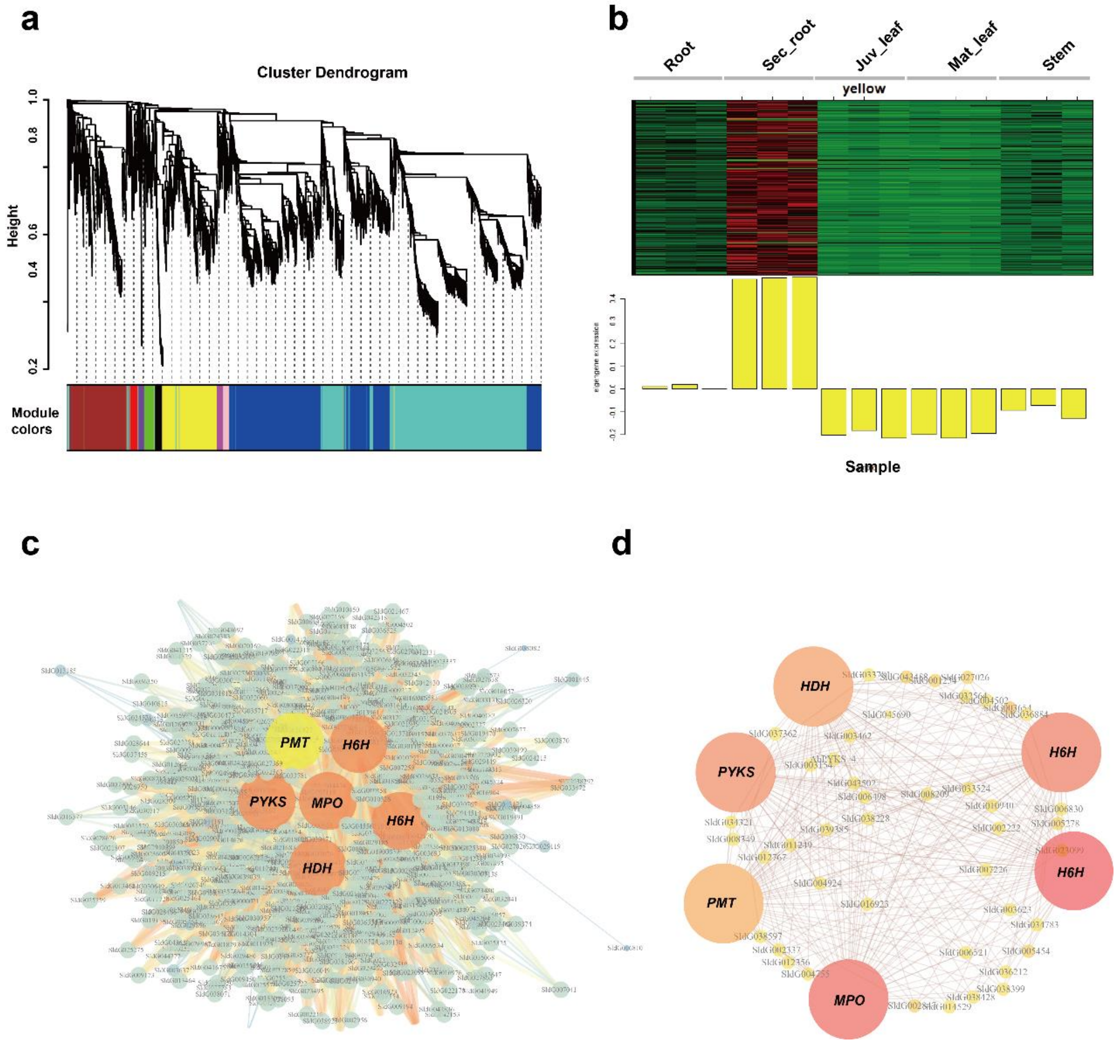

Supplementary Figure 37. The gene regulatory network (GRN) for HS biosynthesis via WGCNA in *A. tanguticus*. a, Hierarchical clustering tree (dendrogram) of genes based on coexpression network analysis based on the transcriptome. b, The display of the secondary root specificity gene expressed value heat map and eigenvalue bar chart are in the yellow module. Each tissue contains three biological replicates. c, Genes related to HS and their interaction partners are displayed in the yellow module with low degree values to small sizes and dark colors. d, Gene regulatory network with degree value ranking top 50 in the yellow module. Source data are provided as a Source Data file.

Supplementary Table 1. The total sequencing data for *A. tanguticus*, *L. chinense, B. arborea* and *M. caulescens.*

Species	Type	Platform	Library type	Reads	Data size (Gb)	Mean read length (bp)	Read N50 (bp)
Anisodus tanguticus	Illumina short reads	HiSeq X (raw)	Paired	844,604,760	126	150	--
		HiSeq X (clean)[1]	Paired	836,871,928	116	150	--
	ONT long reads	Nanopore (raw)	Single	7,566,136	164	21,715	--
		Nanopore (clean)[1]	Single	4,948,922	126	25,709	25,661
	Hi-C reads	HiSeq X (raw)	Paired	1,083,057,122	162.5	150	--
		HiSeq X (clean)[1]	Paired	1,064,114,184	159	150	--
Lycium chinense	Illumina short reads	HiSeq X (raw)	Paired	737,944,473	221	150	--
		HiSeq X (clean)[1]	Paired	709,460,759	212	150	--
	Hi-Fi reads	PacBio (raw)	Single	7,008,932	450	--	--
		PacBio (valid)	Single	3,242,881	54	16,874	16,703
	Hi-C reads	HiSeq X (clean)[1]	Paired	525,935,044	158	150	--
Brugmansia arborea	Illumina short reads	HiSeq X (raw)	Paired	553,477,555	166	150	--
		HiSeq X (clean)[1]	Paired	547,739,659	164	150	--
	Hi-Fi reads	PacBio (raw)	Single	5,875,355	477	--	--
		PacBio (valid)	Single	2,280,393	31	13,645	13,628
	Hi-C reads	HiSeq X (clean)[1]	Paired	377,405,779	113	150	--
Mandragora caulescens	Illumina short reads	HiSeq X (clean)[1]	Paired	347,398,864	104.22	150	--
	Hi-Fi reads	PacBio (valid)	Single	2,169,082	25.01	--	--
	Hi-C reads	HiSeq X (clean)	Paired	273,678,964	129.05	15-	--

[1]The clean data means the raw data after quality control. For HiSeq reads we used fastp software with those commands: -q 20 -5 -3; for long reads we used NextDenovo to perform the error correction and obtain the final consensus sequences with the default parameters.

Supplementary Table 2. Genome estimation results of *A. tanguticus*, *L. chinense*, *B. arborea* and *M. caulescens.*

Species	Software	K-mer	Genome size (bp)	Heterozygous ratio (%)	Repeat ratio (%)
Anisodus tanguticus	Genome Scope	17	1,198,678,208	0.33	48.85
Lycium chinense	Genome Scope	23	1,408,623,534	0.895	53.80
Brugmansia arborea	Genome Scope	17	1,517,262,658	0.642	72.20
Mandragora caulescens	Genome Scope	17	756,690,396	0.05	65.50

Supplementary Table 3. Statistics of the final contig leveled assembly result of *A. tanguticus*, *L. chinense*, *B. arborea* and *M. caulescens* genome.

Species	Statistic type	Contig length (bp)	Contig number
Anisodus tanguticus	N50	23,808,256	21
	Longest	42,618,612	--
	Total contigs	1,249,845,396	205
Lycium chinense	N50	2,994,494	--
	Longest	21,161,401	--
	Total contigs	1,538,392,418	1,406
Brugmansia arborea	N50	7,701,694	--
	Longest	24,794,079	--
	Total contigs	1,548,489,180	1,398
Mandragora caulescens	N50	25,262,060	--
	Longest	33,953,085	--
	Total contigs	711,685,834	808

Supplementary Table 4. The contig cluster of 24 pseudo-chromosomes length of *A. tanguticus*.

Sequences ID	Sequences length	Contig number
LG01	65,099,925	6
LG02	62,018,076	17
LG03	60,155,207	8
LG04	58,322,307	8
LG05	58,220,563	10
LG06	57,859,349	8
LG07	56,911,243	5
LG08	53,678,848	8
LG09	52,682,481	5
LG10	51,350,852	8
LG11	49,959,515	9
LG12	48,764,380	6
LG13	48,486,342	6
LG14	48,430,702	8
LG15	48,267,145	5
LG16	47,781,836	6
LG17	47,759,066	4
LG18	46,779,936	5
LG19	46,547,902	3
LG20	45,262,160	8
LG21	44,456,055	5
LG22	43,267,795	7
LG23	41,002,336	5
LG24	35,127,691	3
Total	1,218,177,812	163
Ratio	97.47%	

Supplementary Table 5. The contig cluster of 12 pseudo-chromosomes length of *L. chinense*.

Sequences ID	Sequences length	Contig number
Chr01	164,731,700	114
Chr02	164,117,743	93
Chr03	141,149,524	84
Chr04	136,104,131	100
Chr05	134,250,335	100
Chr06	132,783,878	111
Chr07	126,893,703	88
Chr08	120,133,093	85
Chr09	102,629,097	249
Chr10	100,053,940	90
Chr11	99,329,694	71
Chr12	94,299,068	72
Total	1,516,475,906	1,257
Ratio	98.57%	

Supplementary Table 6. The contig cluster of 13 pseudo-chromosomes length of *B. arborea*.

Sequences ID	Sequences length	Contig number
Chr01	157,694,634	38
Chr02	135,135,482	51
Chr03	134,161,148	28
Chr04	129,207,246	35
Chr05	125,981,246	46
Chr06	121,782,173	32
Chr07	119,154,430	30
Chr08	113,417,950	23
Chr09	112,782,110	29
Chr10	109,481,803	28
Chr11	102,697,410	21
Chr12	80,663,578	25
Chr13	66,342,360	40
Total	1,508,501,570	426
Ratio	97.42%	

Supplementary Table 7. The contig cluster of 24 pseudo-chromosomes length of *M. caulescens*

Chromosome ID	Chromosome length (bp)
Chr01	38,673,728
Chr02	33,953,085
Chr03	33,343,548
Chr04	32,677,854
Chr05	32,650,972
Chr06	30,127,404
Chr07	30,108,500
Chr08	29,934,184
Chr09	28,822,837
Chr10	28,671,029
Chr11	28,083,551
Chr12	27,725,040
Chr13	27,277,769
Chr14	27,251,986
Chr15	26,251,392
Chr16	25,301,646
Chr17	25,262,060
Chr18	25,128,778
Chr19	25,093,808
Chr20	24,241,081
Chr21	23,456,176
Chr22	23,355,045
Chr23	23,240,880
Chr24	23,100,928
Total	673,733,281
Ratio	94.67%

Supplementary Table 8. Summary of the NGS[1] and transcriptome data and their mapping rate on four species genome assembly.

Species	Type	Library type	Tissue	Total reads number (bp)	Mapped reads number	Mapping rate (%)
Anisodus tanguticus	NGS[1]	Paired	leaf	841,194,056	840,174,753	99.88
	RNA-seq	Paired	root	23,967,220	23,248,203	97.37
	RNA-seq	Paired	stem	23,978,513	23,309,512	97.21
	RNA-seq	Paired	leaf	23,988,965	23,269,296	97.29
Lycium chinense	NGS	Paired	leaf	1,440,100,028	1,422,565,817	98.78
	RNA-seq	Paired	leaf	27,393,766	24,928,327	91.00
	RNA-seq	Paired	stem	29,499,106	27,139,177	92.16
	RNA-seq	Paired	fruit	24,776,256	22,050,867	89.72
	RNA-seq	Paired	root	30,575,956	27,518,360	90.90
Brugmansia arborea	NGS	Paired	leaf	1,100,984,569	1,092,519,569	99.23
	RNA-seq	Paired	leaf	23,686,090	21,791,202	92.86
	RNA-seq	Paired	root	23,726,726	19,218,648	81.10
	RNA-seq	Paired	stem	23,574,090	21,120,027	89.59
Mandragora caulescens	NGS	Paired	All	--	--	99.53
	RNA-seq	Paired	root	23,340,778	22,323,093	95.64
	RNA-seq	Paired	stem	23,699,716	22,823,693	96.30
	RNA-seq	Paired	leaf	23,217,195	22,372,066	96.36

NGS[1], Next genome sequencing.,

Supplementary Table 9. Summary of BUSCO analysis results according to the final contig leveled assembly.

Type	*Anisodus tanguticus*		*Brugmansia arborea*		*Lycium chinense*		*Mandragora caulescens*	
	Number	Compare to the total BUSCO groups (%)	Number	Compare to the total BUSCO groups (%)	Number	Compare to the total BUSCO groups (%)	Number	Compare to the total BUSCO groups (%)
Complete BUSCOs (C)	1,584	98.1	1,584	98.1	1,532	94.9	1,587	98.3
Complete and single-copy BUSCOs (S)	1,124	69.6	1,521	94.2	1,333	82.6	1,359	84.2
Complete and duplicated BUSCOs (D)	460	28.5	63	3.9	199	12.3	228	14.1
Fragmented BUSCOs (F)	13	0.8	13	0.8	18	1.1	15	0.9
Missing BUSCOs (M)	17	1.1	17	1.1	64	4.0	12	0.8
Total BUSCO groups searched	1,614	100	1,614	100	1,614	100	1,614	100

Supplementary Table 10. Gene annotation statistics of the *A. tanguticus* genome assembly.

Gene set		Total genes predicted	Average genes length (bp)	Average CDS length (bp)	Average exon number per gene	Average exon length (bp)	Average intron length (bp)
De novo	Augustus	48,591	4,185.74	1054.53	4.98	211.76	448.60
	Genscan	45,621	15,434.24	1,153.71	6.07	189.95	2,814.60
	Glimmerhmm	47,541	2,019.81	805.71	3.37	238.99	511.99
**Homology**	*Solanum tuberosum*	44,563	4,797.14	1,229.68	5.32	231.22	826.12
	Solanum lycopersicum	42,894	4,940.96	1,247.03	5.43	229.79	834.43
	Capsicum annuum	49,086	4,399.48	1,151.97	5.01	230.05	810.36
	Nicotiana tabacum	56,362	4,088.88	1,109.84	4.76	233.37	793.21
	Solanum pennellii	42,727	4,976.33	1,248.95	5.47	228.39	834.14
	Arabidopsis thaliana	35,897	5,046.36	1,236.68	5.60	220.70	827.58
**RNA-seq**	Trinity+Pasa	22,835	6,909.59	1,183.73	4.82	245.50	942.95
	Tophat+Cufflinks+Pasa	43,607	6,274.34	1,183.73	4.82	245.50	942.95
**Evm**		46,606	2,426.66	1,002.96	3.98	251.73	477.07

Supplementary Table 11. Gene annotation statistics of the *L. chinense* genome assembly.

Gene set		Total genes predicted	Average genes length (bp)	Average CDS length (bp)	Average exon number per gene	Average exon length (bp)	Average intron length (bp)
De novo	August	52,870	4,191.65	1,077.54	4.71	228.80	838.09
	Genscan	49,735	16,680.34	1,085.99	5.57	195.13	3,415.74
	Glimmerhmm	50,643	1,732.64	751.10	2.93	256.41	508.76
**Homology**	*Solanum tuberosum*	39,788	3,921.47	1,234.87	4.71	262.02	723.57
	Solanum lycopersicum	37,547	4,055.22	1,242.18	4.82	257.73	736.44
	Capsicum annuum	45,203	3,578.01	1,128.37	4.44	254.13	712.10
	Nicotiana tabacum	54,925	3,238.71	1,117.96	4.11	272.21	682.58
	Solanum pennellii	37,971	4,054.79	1,240.51	4.86	255.47	729.88
	Arabidopsis thaliana	28,545	4,361.10	1,224.29	5.29	231.63	731.97
**RNA-seq**	Trinity+PASA	36,497	5,120.11	830.10	3.32	473.09	1,531.53
**Evm**		54,946	3,775.37	1,017.84	4.30	236.45	834.45

Supplementary Table 12. Gene annotation statistics of the *B. arborea* genome assembly.

Gene set		Total genes predicted	Average genes length (bp)	Average CDS length (bp)	Average exon number per gene	Average exon length (bp)	Average intron length (bp)
De novo	August	32,283	3,989.29	1,091.69	4.91	222.19	431.12
	Genscan	30,601	25,874.69	1,156.71	5.89	196.42	5,055.91
	Glimmerhmm	32,121	2,007.77	831.05	3.35	248.19	500.97
**Homology**	*Solanum tuberosum*	34,875	4,068.40	1,214.95	4.81	252.67	749.25
	Solanum lycopersicum	32,734	4,247.86	1,232.45	4.92	250.70	770.01
	Capsicum annuum	40,664	3,633.63	1,099.49	4.43	248.25	739.03
	Nicotiana tabacum	45,867	3,388.79	1,087.66	4.26	255.58	706.80
	Solanum pennellii	32,950	4,263.45	1,230.82	4.99	246.58	759.78
	Arabidopsis thaliana	25,164	4626.00	1,237.16	5.30	233.06	786.61
**Evm**		32,347	3,880.88	1,128.02	4.90	230.10	705.45

Supplementary Table 13. Gene annotation statistics of the *M. caulescens* genome assembly.

Gene set		Total genes predicted	Average genes length (bp)	Average CDS length (bp)	Average exon number per gene	Average exon length (bp)	Average intron length (bp)
De novo	Augustus	6,097	4,264.78	6,110.40	27.93	218.77	428.49
	Genscan	24,299	14,991.9	1,420.52	6.81	208.63	2,336.40
	Glimmerhmm	26,106	2,204.03	890.49	3.61	246.70	503.22
**Homolog**	*Solanum tuberosum*	33,568	4,741.68	1,275.99	5.30	240.93	806.73
	Solanum lycopersicum	32,257	4,929.84	1,301.78	5.47	237.78	810.80
	Capsicum annuum	36,284	4,417.51	1,206.07	5.02	240.20	789.66
	Nicotiana tabacum	40,605	4,102.17	1,162.2	4.75	244.83	784.57
	Solanum pennellii	33,099	4,815.23	1,286.93	5.32	241.08	813.30
	Arabidopsis thaliana	28,332	5,013.32	1,265.69	5.57	227.41	820.81
**RNA-seq**	Trinity+PASA	26,985	6,133.52	1,085.47	4.83	224.62	920.17
	EVM	29,193	5,447.13	1,282.79	5.76	222.87	875.65

Supplementary Table 14. Comparison of gene space of the four species genomes with other genomes.

**Species**	**Total genes**	**Average CDS length**	**Average gene length**	**Average exon length**	**Average exons per gene**	**Average intron length**
Anisodus tanguticus	46,606	1,134.68	4,846.05	216.06	5.25	872.93
Lycium chinense	54,946	1,017.84	3,775.38	236.46	4.30	834.45
Brugmansia arborea	32,347	1,128.02	3,880.88	230.10	4.90	705.45
Mandragora caulescens	29,193	1,282.79	5,447.13	222.87	5.76	875.65
Solanum tuberosum	37,885	1,366.75	5,932.47	290.84	6.63	871.45
Solanum lycopersicum	37,534	1,423.99	6,038.58	293.49	7.40	1,522.34

Supplementary Table 15. Summary of BUSCO analysis results according to the annotation of *A. tanguticus, L. chinense*, *B. arborea* and *M. caulescens.*

Type	*Anisodus tanguticus*		*Brugmansia arborea*		*Lycium chinense*		*Mandragora caulescens*	
	Number	Compare to the total BUSCO groups (%)	Number	Compare to the total BUSCO groups (%)	Number	Compare to the total BUSCO groups (%)	Number	Compare to the total BUSCO groups (%)
Complete BUSCOs (C)	1,305	94.9	1,515	93.8	1,487	92.2	1,503	93.1
Complete and single-copy BUSCOs (S)	864	62.8	1,445	89.5	1,276	79.1	1,275	79.0
Complete and duplicated BUSCOs (D)	441	32.1	70	4.3	211	13.1	228	14.1
Fragmented BUSCOs (F)	38	2.8	54	3.3	31	1.9	65	4.0
Missing BUSCOs (M)	32	2.3	45	2.9	96	5.9	46	2.9
Total BUSCO groups searched	1,375	100	1,614	100	1,614	100	1,614	100

Supplementary Table 16. Functional annotation of predicted genes of *A. tanguticus, L. chinense*, *B. arborea* and *M. caulescens.*

	Anisodus tanguticus		*Lycium chinense*		*Brugmansia arborea*		*Mandragora caulescens*	
**Database**	**Number**	**Percent (%)**	**Number**	**Percent (%)**	**Number**	**Percent (%)**	**Number**	**Percent (%)**
**Swiss-Prot**	33,651	72.2	30,747	56.0	23,421	72.4	23,117	79.19
**KEGG**	12,989	27.8	15,198	27.7	12,188	37.7	10,733	36.77
**InterPro**	43,571	93.9	48,611	88.5	30,003	92.8	28,527	97.72
**Pfam**	33,138	71.1	29,117	53.0	23,440	72.5	--	--
**GO**	27,447	59.0	25,289	46.0	19,062	58.9	19,542	66.94
**NR**	42,939	92.1	43,412	79.0	29,828	92.2	27,437	93.98
**COG**	18,387	39.4	16,074	29.3	12,603	38.9	--	--
**Total**	44,784	96.0	50,521	92.0	31,022	95.9	28,773	98.56

Supplementary Table 17. Prediction of repetitive elements in the assembled *A. tanguticus*, *L. chinense*, *B. arborea* and *M. caulescens* genomes.

Type	*A. tanguticus*		*B. arborea*		*L. chinense*		*M. caulescens*	
	Repeat size (Mb)	Perc[1] (%)	Repeat size (Mb)	Perc[1] (%)	Repeat size (Mb)	Perc[1] (%)	Repeat size (Mb)	Perc[1] (%)
RepeatModeler	782	62.57	1,169	75.50	1,025	66.63	485	68.22%
RepeatMasker	331	26.55	551	35.60	316	20.56	162	22.78%
RepeatProtein Masker	280	22.41	304	19.64	235	15.32	104	14.70%
Trf	61	4.90	83	5.40	94	6.12	93	13.20%
Total	820	65.68	1,223	79.0	1,080	70.22	498	70.11%

[1]: Percentage

Supplementary Table 18. Repeat annotation of the *A. tanguticus*, *L. chinense*, *B. arborea* and *M. caulescens* genome.

Type	*Anisodus tanguticus*		*Brugmansia arborea*		*Lycium chinense*		*Mandragora caulescens*	
	Length	Percent (%)	Length	Percent (%)	Length	Percent (%)	Length	Percent (%)
**SINE**	7,858,239	0.63	6,109,519	0.39	5,551,709	0.36	9,501,647	1.33
**LINE**	46,088,697	3.69	41,374,046	2.67	48,531,856	3.15	50,160,624	7.05
**LTR**	538,309,703	43.07	903,821,689	58.37	684,205,373	44.48	260,612,558	36.62
**Copia**	322,198,528	25.78	105,499,359	6.81	65,001,061	4.22	147,072,067	20.67
**Gypsy**	196,073,576	15.69	757,694,010	48.93	605,955,252	39.39	92,227,306	12.96
**DNA**	89,102,372	7.13	105,760,509	6.83	99,857,396	6.49	42,123,546	5.92
**Tandem**	61,271,635	4.90	83,332,776	5.40	94,081,684	6.12	93,910,474	13.20
**Unknown**	134,756,206	10.78	179,784,097	11.61	286,457,169	18.62	117,521,896	16.51
**Total**	820,935,017	65.68	1,223,422,254	79.0	1,080,332,542	70.22	498,981,905	70.11

Supplementary Table 19. Transcription factor identification of *A. tanguticus*, *L. chinense*, *B. arborea* and *M. caulescens* genome.

Species	Type	Number
Anisodus tanguticus	Transcription factors or transcriptional regulators	1,752
	Protein kinase	788
Lycium chinense	Transcription factors or transcriptional regulators	2,884
	Protein kinase	1,581
Brugmansia arborea	Transcription factors or transcriptional regulators	2,325
	Protein kinase	1,110
Mandragora caulescens	Transcription factors or transcriptional regulators	2,727
	Protein kinase	1,177

Supplementary Table 20. Summary of non-protein-coding gene annotations in the *A. tanguticus*, *L. chinense*, *B. arborea* and *M. caulescens* genome assembly.

Species	Software	Type	Number	Average_length
Anisodus tanguticus	tRNAscan-SE	tRNA	3,119	93
	BLAST	rRNA	1,882	200.34
	Infernal	miRNA	269	127.15
		snRNA	1,713	121.39
Lycium chinense	tRNAscan-SE	tRNA	3,781	127
	BLAST	rRNA	12,882	333.19
	Infernal	miRNA	194	121.10
		snRNA	8,845	108.17
Brugmansia arborea	tRNAscan-SE	tRNA	1,916	94
	BLAST	rRNA	7,741	178.37
	Infernal	miRNA	322	123.48
		snRNA	2,204	112.60
Mandragora caulescens	tRNAscan-SE	tRNA	5,619	75.34
	BLAST	rRNA	11,554	651.32
	Infernal	miRNA	142	122.85
		snRNA	859	114.60

Supplementary Table 21. Identity between the closest HS homologue genes amino acid sequence of *A. belladonna* and *A. tanguticus*, *B. arborea and M. caulescens*, respectively. Source data are provided as a Source Data file.

	A. tanguticus	*B. arborea*	*M. caulescens*
***Ab*PMT**	97.6%	88.4%	89.1%
***Ab*PYKS**	97.4%	93.4%	90.8%
***Ab*CYP82M3**	97.1%	81.7%	90.2%
***Ab*TRI**	91.9%	90.4%	90.3%
***AbA*T4**	97.2%	87.1%	92.7%
***Ab*PPAR**	92.4%	85.5%	85.6%
***Ab*UGT1**	96.3%	91.3%	88.1%
***Ab*LS**	92.6%	85.1%	91.6%
***Ab*CYP80F1**	97.3%	88.8%	89.4%
***Ab*HDH**	92.9%	95.6%	91.9%
***Ab*H6H**	84.9%	88.9%	89.2%

Supplementary Table 22. Calculated substitution rate of homologous syntenic gene pairs of *TRIs* between chromosome 2 and 18 of the *A. tanguticus* genome. *K*a, no-synonymous mutation rate; *K*s, synonymous mutation rate.

**Gene pair**	***K*a**	***K*s**	***K*a/*K*s**	**Converted MYA**
Syntenic gene pairs				
SldG043741- SldG045278	0.00214575	0.115811	0.0185281	8.90
SldG043742- SldG045279	0.0692932	0.231081	0.299866	17.78
SldG043743- SldG045280	0.0615942	0.340153	0.181078	26.17
SldG043744- SldG045282	0.151516	0.287002	0.527926	22.08
SldG043745- SldG045283	0.0211507	0.14795	0.142959	11.38
SldG043746- SldG045284	0.0441107	0.14179	0.311099	10.91
SldG043747- SldG045285	0.024174	0.0597864	0.40434	4.59
SldG043748- SldG045286	0.00357591	0.0933416	0.0383099	7.18
SldG043749- SldG045287	0.0401052	0.151947	0.263943	11.69
	95% confidence interval			[7.93, 18.89]

Supplementary Table 23. The results of the ancestral state inference using FastML software.

Source data are provided as a Source Data file.

**FastML**	**Position**	**Probability**
109	V	0.999987
167	V	0.999987

Supplementary Table 24. Gradient profile of mobile phase utilized for UPLC-MS analyses of tropine.

Time (min)	Mobile phase A (%)	Mobile phase B (%)
0	5	95
2	5	95
3	15	85
10	22	78
11	60	40
12	60	40
12.01	5	95
14	5	195

Mobile phase A = 100 mM ammonium formate + 1% formic acid in water. Mobile phase B = acetonitrile

Supplementary Table 25. Kinetic parameters of *At*TRI, *Ba*TRI, *Mc*TRI, *Lc*TRI and *St*TRI. The data represent means of three independent measurements ± SD. The *K*m and *V*max were calculated from the Michaelis-Menten equation with a non-linear regression. The *K*cat value was calculated by dividing *V*max by Et (34kDa) (the number of enzymes in each assay).

Kinetic parameters	*At*TRI	*Ba*TRI	*Mc*TRI	*Lc*TRI	*St*TRI
**pH Assay**	6.4	6.4	6.4	6.4	6.4
***K*m (mM)**	0.21 ± 0.01	0.31 ± 0.03	0.09 ± 0.01	2.91 ± 0.14	n.d.[1]
***V*max (nmol s^{-1} mg^{-1})**	11.53 ± 0.17	59.71 ± 3.04	187.99 ± 2.30	23.82 ± 0.77	n.d.
***K*cat (s^{-1})**	0.34 ± 0.01	1.76 ± 0.09	5.53 ± 0.06	0.70 ± 0.02	n.d.
***K*cat/*K*m**	1.55 ± 0.06	5.60 ± 0.27	61.46 ± 5.76	0.24 ± 0.01	n.d.

Protein abbreviations: *At*TRI: TRI from *A. tanguticus*, *Ba*TRI: TRI from *B. arborea*, *Mc*TRI*:* TRI from *M. caulescens*, *Lc*TRI: TRI from *L. chinense* and *St*TRI: TRI from *S. tuberosum.*

[1]n.d., enzyme reaction not detected.

Supplementary Table 26. Kinetic parameters of wild-type TRI from *S. tuberosum* (*St*TRI) and gain-of-function mutagenesis of *St*TRI. The data represent means of three independent measurements ± SD. The *K*m and *V*max were calculated from the Michaelis-Menten equation with a non-linear regression. The *K*cat value was calculated by dividing *V*max by Et (34kDa) (the number of enzymes in each assay).

**Kinetic parameters**	**Wild-type *St*TRI**	***St*TRI A109V, L167V**	***St*TRI A109V, V155L, L167V, A201G**
**pH Assay**	6.4	6.4	6.4
***K*m(mM)**	n.d.[1]	0.28 ± 0.07	0.11 ± 0.01
***V*max (nmol s^{-1} mg^{-1})**	n.d.	112.82 ± 6.95	128.65± 0.66
***K*cat (s^{-1})**	n.d.	3.31 ± 0.20	3.78 ± 0.02
***K*cat/*K*m**	n.d.	12.42 ± 2.52	35.96 ± 1.61

[1]n.d., enzyme reaction not detected.

Supplementary Table 27. Kinetic parameters of wild-type TRI from *B. arborea* (*Ba*TRI) and loss-of-function site-directed mutagenesis of *Ba*TRI. The data represent means of three independent measurements ± SD. The *K*m and *V*max were calculated from the Michaelis-Menten equation with a non-linear regression. The *K*cat value was calculated by dividing *V*max by Et (34kDa) (the number of enzymes in each assay).

Kinetic parameters	Wild-type *Ba*TRI	*Ba*TRI^{V109A}	*Ba*TRI^{V167L}	*Ba*TRI$^{V109A, V167L}$
**pH Assay**	6.4	6.4	6.4	6.4
***K*m(mM)**	0.31 ± 0.03	0.29 ± 0.05	0.38 ± 0.02	n.d.[1]
***V*max (nmol s^{-1} mg^{-1})**	59.71 ± 3.04	10.75 ± 0.57	7.35 ± 0.11	n.d.
***K*cat (s^{-1})**	1.76 ± 0.09	0.32 ± 0.01	0.22 ± 0.01	n.d.
***K*cat/*K*m**	5.60 ± 0.27	1.12 ± 0.14	0.57 ± 0.03	n.d.

[1]n.d., enzyme reaction not detected.

Supplementary Table 28. Summary of 17 species used for genome synteny analysis

Species	Version	Source
I. nil	Asagao_1.1	https://www.ncbi.nlm.nih.gov/genome/?term=Ipomoea+nil
I. triloba	ASM357664v1	https://www.ncbi.nlm.nih.gov/genome/?term=Ipomoea+triloba
P. axillaris	v1.6.2	https://solgenomics.net/organism/Petunia_axillaris/genome
P. inflata	v1.0.1	https://solgenomics.net/organism/Petunia_inflata/genome
N. attenuata	NIATTr2	NCBI
S. melongena	v4.1	http://eggplant.kazusa.or.jp/
S. lycopersicum	build_4.00	https://solgenomics.net/ftp/genomes/
S. tuberosum	Stuberosum_v6.1	https://solgenomics.net/ftp/genomes/
P. floridana	--	https://ngdc.cncb.ac.cn/search/?dbId=gwh&q=Physalis%20floridana&page=1
I. cyaneum	v1.0	https://solgenomics.net
C. annuum	Zunla-1	https://www.ncbi.nlm.nih.gov/genome/?term=Capsicum+annuum
C. chinense	--	https://www.ncbi.nlm.nih.gov/Traces/wgs/?val=MCIT02#contigs
C. baccatum	--	https://www.ncbi.nlm.nih.gov/Traces/wgs/?val=MLFT02#contigs
A. tanguticus	--	This study
B. arborea	--	This study
L. chinense	--	This study
M. caulescens	--	This study

Supplementary Table 29. Primers used for TRIs constructing protein expression plasmids.

Primer name		Sequence (5' to 3')
*At*TRI-F	Forward primer	AATGGGTCGCGGATCCATGGGAGAATCAAAAGTTTACATGA
*At*TRI-R	Reverse primer	GGTGGTGGTGCTCGAGAAACCCACCATTAGCTGTGA
*Ba*TRI-F	Forward primer	GGTGGTGGTGCTCGAGATGGAAGAATCAAAAGATAACATGAATGGCAAC
*Ba*TRI-R	Reverse primer	AATGGGTCGCGGATCCAAACCCACCATTAGCTGTGAATCC
*Mc*TRI-F	Forward primer	GGATCCATGGAAGAATCAAAAGT
*Mc*TRI-R	Reverse primer	GAGCTCTTAAAACCCACCATTAGC
*Lc*TRI-F	Forward primer	AATGGGTCGCGGATCCATGACAGAATCAAACGACAACG
*Lc*TRI-R	Reverse primer	GGTGGTGGTGCTCGAGAAATCCATTAGCAGTGAATCCA
*St*TRI-F	Forward primer	AATGGGTCGCGGATCCATGGCAGAATTGAGAGAAAAAT
*St*TRI-R	Reverse primer	GGTGGTGGTGCTCGAGAAACCCACCATTAGCTGTAAA
*Ba*TRI-V109A-F	Forward primer for constructing protein expression plasmid of *Ba*TRI-V109A	ACGCAGGGGTGGCGATACATAAGG
*Ba*TRI-V109A-R	Reverse primer for constructing protein expression plasmid of *Ba*TRI-V109A	CCTTATGTATCGCCACCCCTGCGT
*Ba*TRI-V167L-F	Forward primer for constructing protein expression plasmid of *Ba*TRI-V167L	GCACTGCCTTCGCTTTCTCTTTATTCTGC
*Ba*TRI-V167L-R	Reverse primer for constructing protein expression plasmid of *Ba*TRI-V167L	GCAGAATAAAGAGAAAGCGAAGGCAGTGC
*St*TRI-A109V-F	Forward primer for constructing protein expression plasmid of *St*TRI-A109V	GCAGGAGTGGTAATACATAAGGAAG
*St*TRI-A109V-R	Reverse primer for constructing protein expression plasmid of *St*TRI-A109V	CTTCCTTATGTATTACCACTCCTGC
*St*TRI-V155L-F	Forward primer for constructing protein expression plasmid of *St*TRI-V155L	GGAAATGTTATTTTTCTTTCTTCTATTGC
*St*TRI-V155L-R	Reverse primer for constructing protein expression plasmid of *St*TRI-V155L	GCAATAGAAGAAAGAAAAATAACATTTCC
*St*TRI-L167V-F	Forward primer for constructing protein expression plasmid of *St*TRI-L167V	GCATTGCCCTCTGTTTCTCTTTACTC
*St*TRI-L167V-R	Reverse primer for constructing protein expression plasmid of *St*TRI-L167V	GAGTAAAGAGAAACAGAGGGCAATGC
*St*TRI-A201G-F	Forward primer for constructing protein expression plasmid of *St*TRI-A201G	CTGTTGCTCCAGGAGTCATTTTAACC
*St*TRI-A201G-R	Reverse primer for constructing protein expression plasmid of *St*TRI-A201G	GGTTAAAATGACTCCTGGAGCAACAG

Supplementary Table 30. Primers used for LSs cloning and constructing overexpression plasmids.

Primer name		Sequence (5' to 3')
AtLS-F	Forward primer for cloning *At*LS	ATGAAGAAATCAATTGTGGTTCCA
AtLS -R	Reverse primer for cloning *At*LS	TTATAGAGGTTCATAATATATCCATCT
*Ba*LS-F	Forward primer for cloning *Ba*LS	ATGAGGAATTCCATCTTGAACTACCA
*Ba*LS-R	Reverse primer for cloning *Ba*LS	CTATAGAGATTCATTAGATATCCATCT
*At*LS-inpEAQ-F	Forward primer for constructing overexpression plasmid of *At*LS	CAAATTCGCGACCGGTATGAAGAAATCAATTGTGGTTCCA
*At*LS-inpEAQ-R	Reverse primer for constructing overexpression plasmid of *At*LS	AGTTAAAGGCCTCGAGTTATAGAGGTTCATAATATATCCATCT
*Ba*LS-inpEAQ-F	Forward primer for constructing overexpression plasmid of *Ba*LS	CAAATTCGCGACCGGTATGAGGAATTCCATCTTGAACTACCA
*Ba*LS-inpEAQ-R	Reverse primer for constructing overexpression plasmid of *Ba*LS	AGTTAAAGGCCTCGAGCTATAGAGATTCATTAGATATCCATCT
*Ab*LS-inpEAQ-F	Forward primer for constructing overexpression plasmid of *Ab*LS	CTGCCCAAATTCGCGACCGGTATGAAGAAAACAATTGTGGTTCC
*Ab*LS-inpEAQ-R	Reverse primer constructing overexpression plasmid of *Ab*LS	ACCAGAGTTAAAGGCCTCGAGTTATAGAGGTTGATAATATATCCA
*Ab*UGT1-inpEAQ-F	Forward primer for constructing overexpression plasmid of *Ab*UGT1	ATGGAAGAATCAAAAGTGTCCATGATGAATTG
*Ab*UGT1-inpEAQ-R	Reverse primer constructing overexpression plasmid of *Ab*UGT1	CAAATTCGCGACCGGTATGGGCTTAGGACATGTCAATCCA

Supplementary Table 31. Primers used for PPARs constructing protein expression plasmids.

Primer name		Sequence (5' to 3')
*Pi*PPAR-F	Forward primer	GAA TTC ATGGGGGAACAATCTGATGAACA
*Pi*PPAR -R	Reverse primer	GTC GAC TCACTCCAGGTGGGTGACTG
*Ba*PPAR-F	Forward primer	GGA TCC ATGGGTGATTGCAGGAGCTA
*Ba*PPAR-R	Reverse primer	GAG CTC TCAGACAGTAGTAGTAGTAGTGCA
*Mc*TRI-F	Forward primer	GGA TCC ATGGGTGATTGCAGGAGCTA
*Mc*TRI-R	Reverse primer	GTC GAC TCAAACTCCATGCATAACTGGTG

Supplementary Table 32. Primers used for CYP80F1 and HDH constructing overexpression plasmid.

Primer name		Sequence (5' to 3')
*Pi*CYP80F1-F	Forward primer	CAAATTCGCGACCGGT<br>ATGGCACAGGATTTATTTGTAGCG
*Pi*CYP80F1 -R	Forward primer	AGTTAAAGGCCTCGAG<br>TTATCTTCTAGTTTTTAGCCATAGCAC
*At*CYP80F1-F	Forward primer	ACCGGTATGAATATTGAAAACACGA
*At*CYP80F1-R	Reverse primer	CTCGAGTTATGACTTCCTCA
*Ba*CYP80F1-F	Forward primer	ACC GGT ATGAATATTGGAAACACAACATTTGA
*Ba*CYP80F1-R	Reverse primer	CTC GAG TTATTCTATTAATTTTCTCATTTTTGG
*Mc*CYP80F1-F	Forward primer	ACC GGT ATGAATATTGAAAACACAATATTCGA
*Mc*CYP80F1-R	Forward primer	CTC GAG TTATTTCCTCAGTTTTGGTATGACA
*At*HDH-F	Forward primer	ACCGGTATGGCTTCTGAGAA
*At*HDH-R	Forward primer	CTCGAGCTAGGTTTGAGCAG
*Ba*HDH-F	Forward primer	ACC GGT ATGGCTGTTGAGAAATTATCAGA
*Ba*HDH-R	Reverse primer	CTC GAG CTAGGTTTGAACAGCAATCAATGT
*Mc*HDH-F	Forward primer	ACC GGT ATGGATTCCTCTGGTGTCCTC
*Mc*HDH-R	Reverse primer	CTC GAG TTATATGATGCTGGTTTTAGCAGGA
*It*HDH-F	Forward primer	ACCGGTATGGCAGAAAATTCA
*It*HDH-R	Forward primer	CTCGAGTTAAGCAGAATTTAATGTGT
*In*HDH-F	Forward primer	ACCGGTATGGGCTCGGAATC
*In*HDH-R	Forward primer	CTCGAGTTATGCCGGTGGAG

Supplementary Table 33. Primers used for H6Hs constructing protein expression plasmids.

Primer name		Sequence (5' to 3')
*Mc*H6H-F	Forward primer	GGA TCC ATGGCTACTTTAATCTCAAATTGGTGT
McH6H -R	Reverse primer	GAG CTC TTAGGCATTGATTTTATATGGCT
LcH6H-F	Forward primer	GGA TCC ATGGCATCTCTCATCTCTAGCTG
LcH6H -R	Reverse primer	GTC GAC TTAAATTTTATATGGATTGAGTGCAGC

Supplementary Table 34. Oligonucleotide primers used in this study.

Primer	Sequence (5´-3´)	Use
*At*PDS VF	CGTCTAGAGGATCTTGTTTAGTGTTTAGGTGA	VIGS construct assembly
*At*PDS VR	GCGGATCCTAACAGCAGAAACAAGTCCAAT	VIGS construct assembly
*At*TRI VF	CGTCTAGATTCTTATATTACTGGCCAGATCA	VIGS construct assembly
*At*TRI VR	GCGGATCCTAGCTGACACAAGAATCATCTAGG	VIGS construct assembly
*At*LS VF	CGTCTAGACATAAAGGGTGCAGGACATAC	VIGS construct assembly
*At*LS VR	GCGGATCCAAATCTCAATACAGTGAAACATATTT	VIGS construct assembly
*At*HDH VF	CGTCTAGACATTGGTTGCTGCTCAAAC	VIGS construct assembly
*At*HDH VR	GCGGATCCTTTTTCATATCAACCGCATG	VIGS construct assembly
*At*H6H VF	CGTCTAGAAAAATGTCAAAAATCAGTAAGTAAA	VIGS construct assembly
*At*H6H VR	GCGGATCCGACAAGAGTAGCCATCAAAAGTC	VIGS construct assembly
*At*PGK VF	TTTGCTGCCATTGTTGGTGG	qRT-PCR
*At*PGK VR	CGTACCCTTGGGCCTTGTAG	qRT-PCR
*At*TRI VF	CCACTGCCCTTGTTACTGGT	qRT-PCR
*At*TRI VR	TGTATCACCACCCCTGCATT	qRT-PCR
*At*LS VF	TCCAGAAGTTGACCCCCTCA	qRT-PCR
*At*LS VR	TTTGCGACCTTGGTGTACGA	qRT-PCR
*At*HDH VF	ACTTTGGGATGGGCAGCTAT	qRT-PCR
*At*HDH VR	TCCCACCTCTGTTGCTATGC	qRT-PCR
*At*H6H VF	ACAACTCTCTAACGGGGAGC	qRT-PCR
*At*H6H VR	ATGTTGCTGGCTTTTCAGGC	qRT-PCR

Supplementary Table 35. Gradient profile of mobile phase for UPLC-MS analyses of littorine, hyoscyamine, anisodamine and scopolamine. Mobile phase A = 0.1% formic acid in water. Mobile phase B = Acetonitrile.

Time (min)	Mobile phase A (%)	Mobile phase B (%)
0	99	1
2	99	1
4.5	85	15
7	75	25
8.5	50	50
9	5	95
10	2	98
11	99	1
12	99	1

Supplementary Table 36. UPLC mobile phase gradients utilized for LC-MS/MS analyses of phenyllactate. Mobile phase A: 0.1% formic acid in water, mobile phase B: acetonitrile

Time (min)	Mobile phase A (%)	Mobile phase B (%)
0	98	2
1	98	2
6.5	2	98
7.5	98	2
10	98	2

Supplementary references

1. Jin, J.J. *et al*. GetOrganelle: a fast and versatile toolkit for accurate de novo assembly of organelle genomes. *Genome Biol.* **21,** 241 (2020).
2. Kazutaka, K. MAFFT: a novel method for rapid multiple sequence alignment based on fast Fourier transform. *Nucleic Acids Res.* **14,** 3059-3066 (2002).
3. Alexandros, S. RAxML-VI-HPC: maximum likelihood-based phylogenetic analyses with thousands of taxa and mixed models. *Bioinformatics* **21,** 2688-2690 (2006).
4. Li, L., Stoeckert, C.J. & Roos, D.S. OrthoMCL: Identification of ortholog groups for eukaryotic genomes. *Genome Res.* **13,** 2178-2189 (2003).
5. Langfelder, P. & Horvath, S. WGCNA: an R package for weighted correlation network analysis. *BMC Bioinformatics* **9,** 559 (2008).
6. Langfelder, P. & Horvath, S. Eigengene networks for studying the relationships between co-expression modules. *Bmc Syst. Biol.* **1,** 54 (2007).
7. Peter, L., Mischel, P.S., Steve, H. & Timothy, R. When is hub gene selection better than standard meta-analysis? *Plos One* **8,** 61505 (2013).
8. Shannon, P. *et al*. Cytoscape: a software environment for integrated models of biomolecular interaction networks. *Genome Res.* **13**, 2498-2504 (1971).
