# Supplementary material for: Multiple independent losses of the biosynthetic pathway for two tropane alkaloids in the Solanaceae family
Source: Nat Commun. 2023 Dec 20;14:8457. doi: 10.1038/s41467-023-44246-3 (PMC10730914; doi:10.1038/s41467-023-44246-3)
Supplement: Supplementary file 3 — Description of Additional Supplementary Files [file 41467_2023_44246_MOESM3_ESM.pdf]

Description of Additional Supplementary Files

File Name: Supplementary Data 1
Description: Evaluation of three species genome completeness using data set of RNA transcripts.
