# Supplementary material for: Multiple independent losses of the biosynthetic pathway for two tropane alkaloids in the Solanaceae family
Source: Nat Commun. 2023 Dec 20;14:8457. doi: 10.1038/s41467-023-44246-3 (PMC10730914; doi:10.1038/s41467-023-44246-3)
Supplement: Supplementary file 5 — Reporting Summary [file 41467_2023_44246_MOESM5_ESM.pdf]

nature portfolio

Corresponding author(s): Jianquan Liu

Last updated by author(s): Nov 30, 2023

Reporting Summary

Nature Portfolio wishes to improve the reproducibility of the work that we publish. This form provides structure for consistency and transparency in reporting. For further information on Nature Portfolio policies, see our Editorial Policies and the Editorial Policy Checklist.

Statistics

For all statistical analyses, confirm that the following items are present in the figure legend, table legend, main text, or Methods section.

n/a	Confirmed	
☐	☒	The exact sample size (*n*) for each experimental group/condition, given as a discrete number and unit of measurement
☒	☐	A statement on whether measurements were taken from distinct samples or whether the same sample was measured repeatedly
☐	☒	The statistical test(s) used AND whether they are one- or two-sided<br>*Only common tests should be described solely by name; describe more complex techniques in the Methods section.*
☒	☐	A description of all covariates tested
☐	☒	A description of any assumptions or corrections, such as tests of normality and adjustment for multiple comparisons
☐	☒	A full description of the statistical parameters including central tendency (e.g. means) or other basic estimates (e.g. regression coefficient) AND variation (e.g. standard deviation) or associated estimates of uncertainty (e.g. confidence intervals)
☒	☐	For null hypothesis testing, the test statistic (e.g. *F*, *t*, *r*) with confidence intervals, effect sizes, degrees of freedom and *P* value noted<br>*Give P values as exact values whenever suitable.*
☒	☐	For Bayesian analysis, information on the choice of priors and Markov chain Monte Carlo settings
☒	☐	For hierarchical and complex designs, identification of the appropriate level for tests and full reporting of outcomes
☒	☐	Estimates of effect sizes (e.g. Cohen's *d*, Pearson's *r*), indicating how they were calculated

Our web collection on statistics for biologists contains articles on many of the points above.

Software and code

Policy information about availability of computer code

Data collection	No software was used to collect the data
Data analysis	Genome size estimation: GenomeScope v2.0 and Jellyfish v2.3.0 Genome assembly: Nextdenovo vl.1.1, Nextpolish vl.2.0, Pilon vl.23, hifiasm v0.12, Bowtie2 v2.3.0 and LACHESIS vl.O Genome assessment: bwa v0.7.12-r1039, Trinity v2.8.4 and BUSCO v3.0 Genome annotation: Tandem Repeats Finder v4.04, RepeatMasker v4.0.7, RepeatModeler vl.0.11, BLASTP v2.3.0, PASA v2.3.3, AUGUSTUS v3.2.3, Genscan vl.O, GlimmerHMM v3.0.4, GeMoMa vl.6.1, EvidenceModeler vl.1.1, tRNAscan-SE v2.0, INFERNAL vl.O, lnterProScan vS.36-75.0 Transcriptome analysis: Trinity v2.8.4, Cufflinks v2.2.l, PASA v2.3.3, HISAT2 v2.2.l, StringTie v2.l.3, WGCNA vl.68, Cytoscape v3.6.0 Phylogenetic analyses: OrthoMCL v2.0.9, MAFFT v7.402, RAxML v8.2.12Gene family analysis: CAFE v4.2 Divergence time estimation: MCMCTree in the PAML package v4.9h Polyploidzation analysis: MCScan vl.2.7, MCScanX vl, WGDI v0.4.1 Hyoscyamine and scopolamine (HS) synthesis genes identify: BLASTP v2.3.0, HMMER v3.3, lnterProScan vS.36-75.0 Microsynteny analysis: BLASTP v2.3.0, JCVI (MCScan python-ve[illegible]sion vl.2.7), LAST v2.32.l The enzyme kinetic constants calculation: OriginPro v9.l The tertiary structure of protein construction: PyMOL v2.2.0 Inferences of ancestral states: FastML v3.ll

For manuscripts utilizing custom algorithms or software that are central to the research but not yet described in published literature, software must be made available to editors and reviewers. We strongly encourage code deposition in a community repository (e.g. GitHub). See the Nature Portfolio guidelines for submitting code & software for further information.

Data

Policy information about availability of data

All manuscripts must include a data availability statement. This statement should provide the following information, where applicable:

- Accession codes, unique identifiers, or web links for publicly available datasets
- A description of any restrictions on data availability
- For clinical datasets or third party data, please ensure that the statement adheres to our policy

All genomic sequencing data and transcriptomic raw data used in this study have been deposited in the NCBI Sequence Read Archive (SRA) under BioProject accession numbers PRJNA765943 for Anisodus tanguticus [https://dataview.ncbi.nlm.nih.gov/object/PRJNA765943], PRJNA765960 for Brugmansia arborea [https://dataview.ncbi.nlm.nih.gov/object/PRJNA765960], PRJNA765963 for Lycium chinense [https://dataview.ncbi.nlm.nih.gov/object/PRJNA765963] and PRJNA903289 for Mandragora caulescens [https://dataview.ncbi.nlm.nih.gov/object/PRJNA903289]. The four species genome assembly and annotations are also available at Figshare with https://figshare.com/s/ecd289d0edea48c839b0 (Anisodus tanguticus), https://figshare.com/s/91c3ad78a30fed3fcdb4 (Brugmansia arborea), https://figshare.com/s/9b601f69085726ab3b2a (Lycium chinense) and https://figshare.com/s/b95148b8ae0d72fb7f0f (Mandragora caulescens).

Research involving human participants, their data, or biological material

Policy information about studies with human participants or human data. See also policy information about sex, gender (identity/presentation), and sexual orientation and race, ethnicity and racism.

Reporting on sex and gender	not applicable
Reporting on race, ethnicity, or other socially relevant groupings	not applicable
Population characteristics	not applicable
Recruitment	not applicable
Ethics oversight	not applicable

Note that full information on the approval of the study protocol must also be provided in the manuscript.

Field-specific reporting

Please select the one below that is the best fit for your research. If you are not sure, read the appropriate sections before making your selection.

☒ Life sciences ☐ Behavioural & social sciences ☐ Ecological, evolutionary & environmental sciences

For a reference copy of the document with all sections, see nature.com/documents/nr-reporting-summary-flat.pdf

Life sciences study design

All studies must disclose on these points even when the disclosure is negative.

Sample size	For genome sequencing, one mature individual plant is sufficient from each species. For RNA sequencing, three replications for different tissues (leaf, root, secondary root and stem) from A. tanguticus, B. arborea and M. caulescens individuals were also collected, and used for transcriptome sequencing. For L chinense, four tissues were used for RNA-sequencing: root, fruit, stem and leaf (each in triplicate). For enzyme activity analysis, three independent replicate assays are commonly used for enzymatic reaction assays according to a large number of literature reports. Therefore, we implemented three independent reaction tests for each substrate concentration and performed a two-tailed t-test test on the results. For the virus-induced gene silencing (VIGS) experiments, six to eight biological replicates were usually conducted based on a large number of peer-reported experimental data, and we performed six replicates and then performed a two-tailed t-test test on the results. We have described the sample size and the statistical method in each individual experiment.
Data exclusions	For the long reads, we filtered the low-quality (mean_qscore < 7) reads, and then further corrected by Nextdenovo (read_cutoff=2k,seed_cutoff=30k, blocksize=l.Sg). For the Illumina short reads the following criteria were performed to filter the low quality reads: (i) containing more than 5% unidentified nucleotides, (ii) more than 65% of bases with a Phred quality score< 7, and (iii) more than 10 bp adapter sequences (allowing 2 bp mismatches)
Replication	To avoid affecting genome assembly, we selected one individual of four plant genome sequencing, respectively. therefore, no replication was performed. All the tissues for RNA sequencing have three replications and these attempts at replication were successful. For the enzyme activity experiments, three independent reactions were tested for each substrate concentration, and all three independent replicates were performed successively in the same period. For the virus-induced gene silencing (VIGS) experiments, six independent expression down-regulated lines obtained at the same time were used for the alkaloid measurement. All these attempts at replication were successful. We have described the number of replication in each individual experiments.
Randomization	There were no filed or lab experiment that might require randomization. For the virus-induced gene silencing (VIGS) experiments, only PCR-

Randomization

positive and down-regulated expression strains were used for alkaloid measurements. For TRI and other enzyme activity experiments, different enzymes require different substrate concentrations. In order to simulate as perfect a Michaelis-Menten curve as possible (reflecting the ascending and plateau phases),the range of kinetic tests for each enzyme is experimentally explored. The kinetic constants of the enzyme is calculated from the curve, so this experiment cannot be randomization.

Blinding

Blinding is not applicable in our study because it does not involve subjects which receive different treatments.

Reporting for specific materials, systems and methods

We require information from authors about some types of materials, experimental systems and methods used in many studies. Here, indicate whether each material, system or method listed is relevant to your study. If you are not sure if a list item applies to your research, read the appropriate section before selecting a response.

Materials & experimental systems

n/a	Involved in the study
☒	☐ Antibodies
☒	☐ Eukaryotic cell lines
☒	☐ Palaeontology and archaeology
☒	☐ Animals and other organisms
☒	☐ Clinical data
☒	☐ Dual use research of concern
☐	☒ Plants

Methods

n/a	Involved in the study
☒	☐ ChIP-seq
☒	☐ Flow cytometry
☒	☐ MRI-based neuroimaging

Plants

Seed stocks

Materials for high-quality sequencing and seeds for other experiments were collected in wild at high altitudes. These individual of A. tanguticus, B. arboream, M. caulescens, and L. chinense are from Menyuan (Qinghai Province, voucher specimens Liujq201904), Chengdu (Sichuan Province, Liujq201905), Guoluo (Qinghai Province, Liujq202203), and Lanzhou (Gansu Province, Liujq202005), China.

Novel plant genotypes

We obtained transient transgenic lines of TRI, LS, HDH and H6H genes by virus-induced gene silencing (VIGS) technology as described in methods. The six independent expression down-regulated lines were used for the alkaloid measurement. Because of the instantaneous transformation, there is no generation reproduction. T-DNA insertion and Cas9 editing techniques are not used.

Authentication

N/A
